# Fundamentals of Crystalline Evolution and Properties of Carbon Nanotube-Reinforced Polyether Ether Ketone Nanocomposites in Fused Filament Fabrication

**DOI:** 10.1021/acsami.3c01307

**Published:** 2023-04-26

**Authors:** Mia Carrola, Hamed Fallahi, Hilmar Koerner, Lisa M. Pérez, Amir Asadi

**Affiliations:** † Department of Materials Science & Engineering, Texas A&M University, College Station, Texas 77843, United States; ‡ Department of Mechanical Engineering, Texas A&M University, College Station, Texas 77843, United States; § Materials & Manufacturing Directorate, Air Force Research Laboratory, WPAFB, Dayton, Ohio 45430, United States; ∥ High Performance Research Computing, Texas A&M University, MS 3361, College Station, Texas 77843-3361, United States; ⊥ Department of Engineering Technology & Industrial Distribution, Texas A&M University, College Station, Texas 77843, United States

**Keywords:** polyetheretherketone, nanocomposites, carbon nanotubes, fused filament fabrication, cold crystallization

## Abstract

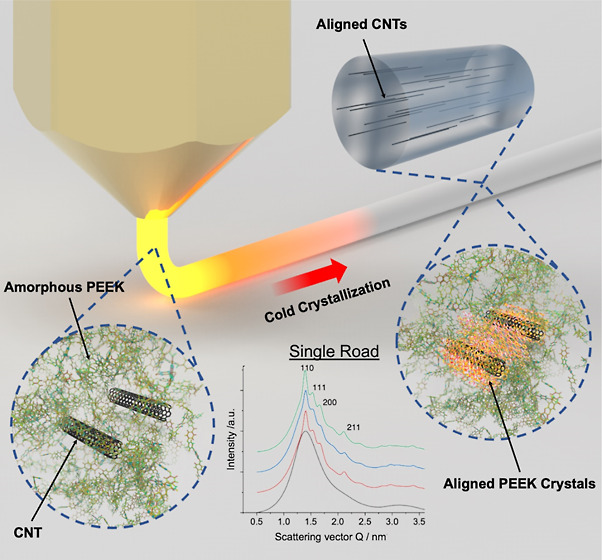

As fused filament fabrication (FFF) continues to gain popularity, many studies are turning to nanomaterials or optimization of printing parameters to improve the materials’ properties; however, many overlook how materials formulation and additive manufacturing (AM) processes cooperatively engineer the evolution of properties across length scales. Evaluating the in-process evolution of the nanocomposite using AM will provide a fundamental understanding of the material’s microstructure, which can be tailored to create unique characteristics in functionality and performance. In this study, the crystallinity behavior of polyetheretherketone (PEEK) was studied in the presence of carbon nanotubes (CNTs) as a nucleation aid for improved crystallization during FFF processing. Using various characterization techniques and molecular dynamics simulations, it was discovered that the crystallization behavior of extruded filaments is very different from that of 3D printed roads. Additionally, the printed material exhibited cold crystallization, and the CNT addition increased the crystallization of printed roads, which were amorphous without CNT addition. Tensile strength and modulus were increased by as much as 42 and 51%, respectively, due to higher crystallinity during printing. Detailed knowledge on the morphology of PEEK–CNT used in FFF allows gaining a fundamental understanding of the morphological evolution occurring during the AM process that in turn enables formulating materials for the AM process to achieve tailored mechanical and functional properties, such as crystallinity or conductivity.

## 1 Introduction

Additive manufacturing (AM) has garnered exponential popularity over the past three decades, which provides a method to manufacture intricate, custom parts and offer rapid prototyping capabilities. Fused filament fabrication (FFF), also commonly referred to as “3D printing”, is an AM method which works based on layer-by-layer stacking of extruded polymer filaments. The ease of use and possibility of creating complex topologies make the FFF process a very attractive option for a multitude of industries to use for applications that include—but are not limited to—medical implants, construction, and tooling for vehicular components.^1−4^ Though the process offers many appealing features in forming thermoplastics, FFF does have several challenges that still need to be addressed; the most important of which are the low mechanical properties of printed parts.^5,6^ This is due to the weak interlayer adhesion between the printed layers and the porosity of produced parts, each accelerating delamination and catastrophic failure of 3D printed parts. Often, commodity polymers such as poly(lactic acid) (PLA) and acrylonitrile butadiene styrene (ABS) are selected as filament feedstocks due to their low cost, despite their limited mechanical properties and low operating temperatures.

A class of high-performance polymers have emerged with superior characteristics to the aforementioned commodity polymers, known as the polyaryletherketone (PAEK) polymer family. This family of polymers, occasionally referred to as the “aromatic polyketones”, includes polymers that consist of at least one aryl, one ether, and one ketone building block. The semi-crystalline PAEKs have properties that include good fatigue and wear resistance, excellent strength and stiffness, and chemical resistance that are ideal for high-end in-service applications.^7^ This family has also been shown to fit well into novel AM technologies.^8^ Among semi-crystalline PAEK polymers, the most commercially successful polymer is polyetheretherketone (PEEK). PEEK shows unrivaled properties including impressive mechanical stiffness and strength, chemical stability, and heat resistance. In some instances, the polymeric material aims to replace metal components, allowing for lightweight structures in several industries including textiles, automotive, and aerospace. One of the limitations of PEEK is its fast crystallization kinetics during cooling from the melt, which leads to reproducibility issues in processing, including AM.^9^

Semi-crystalline polymers can form densely packed, ordered regions with a lamellar morphology, denoted as crystalline regions. These regions are embedded in and connected to disordered, amorphous regions. Crystalline regions provide higher thermal stability and chemical resistance, as well as increased strength, stiffness, and wear resistance.^10^ Amorphous regions are associated with a higher impact resistance. Crystallization has been shown to be controllable through careful selection of processing parameters and post processing treatments.^11^ During the FFF process, orientational melt memory is maximum in the outer shell of the extrudate, which later will form the interfacial region for the adjacent layer.^12^ The poor mechanical performance of 3D-printed parts mainly stems from weak adhesion between successively deposited roads. The formation of a good interlayer region is governed by molecular diffusion between the extruded road shells, which consists of five steps: surface rearrangement, surface approach, wetting, diffusion, and randomization.^13^ The interlayer bonding quality is highly defined by the extent of chain diffusion between the adjacent layers. Orientational effects occurring during the FFF process and chain mobility reduction in the cooled substrate layer impede thorough molecular diffusion in the build direction. Furthermore, the crystallinity of the extrudate shell lowers the chance of chain inter-diffusion and entanglement during the interlayer formation process. In addition to weak bonding strength, high residual stresses across the interlayer region make this zone the most favorable place for cracks to form and grow.

Carbon nanomaterials, most commonly graphene and carbon nanotubes (CNTs), have been widely used with PEEK and shown to improve the mechanical properties and add functionality to the material.^14^ CNTs, specifically, have been widely adopted and studied due to their exceptional mechanical and physical features, such as high strength and stiffness.^15^ Although further studies are needed, research suggests that the presence of CNTs in PEEK could have two opposing effects, namely a heterogeneous nucleation effect and a confinement effect.^16^ The former increases the crystallization temperature *T*_c_ and provides a high specific surface for polymer chains to generate ordered structures, and the latter influences decreases in *T*_c_ due to the restricting effect of CNTs on polymer chain mobility.^17^ Hence, CNTs can be useful in effectively engineering the semi-crystalline morphology and establishing relationships between processing strategies and the resultant material properties. An important aspect of studying semi-crystalline systems having CNTs is the promoted crystallization due to the large surface area that CNT offers for polymer chains, which act as nucleation sites.^18,19^ CNTs have been shown to induce crystallinity even in amorphous polymers that do not crystallize under normal conditions.^20^

Several studies have combined the features of PEEK and CNTs to improve the properties and functionality of manufactured parts.^21−23^ Other studies focused on the thermal parameters during the processing (heat treatment, nozzle temperature, and ambient temperature) or other printing parameters (deposition pattern and thickness, infill density, and nanomaterial content) and the resulting effects on the material properties and microstructure of the printed parts.^24−27^ However, there are still discrepancies between different reports and their conclusions.^17,28^ This is partly due to the process-sensitive nature of semi-crystalline thermoplastics and their nanocomposites and especially true for PEEK due to its fast crystallization kinetics. There are relatively few attempts to focus on the governing phenomena occurring during the extrusion-based AM of PEEK nanocomposites. Often times, the emphasis of these studies is on improving the mechanical properties and material characteristics, without determining the foundational mechanisms that contribute to these improvements. To justify the current discrepancies and establish the manufacturing science of 3D-printing PEEK–CNT systems, more systematic investigations are required, which consist of tracking the crystal morphology in the nanocomposite feedstock and its changes during the subsequent cold crystallization. The emergence of in-operando techniques, such as X-ray photon correlation spectroscopy (XPCS), allows for the study of the extrusion dynamics in real time and to gain knowledge of the characteristic behaviors of the material at the nanoscale.^29^ However, these methods are incapable of capturing molecular interactions at the atomic scale. Using molecular dynamics (MD) simulations, several studies have reported the formation of organized chain structures around the CNTs for polyimide and polyethylene matrices.^30,31^ Exploring the atomic scale changes in the matrix surrounding the CNTs is essential to understand the molecular mechanisms, which will aid in controlling material properties. It should be noted that PEEK and its nanocomposites exhibit hierarchical structures with a characteristic length scale of several microns.^32^ Current fully atomistic MD simulation techniques are limited to orders of nanometers and are unable to effectively model all the features spanning the entire micron-scale length scales.^33^ Despite this, they offer beneficial insights into the initial phases of matrix crystal nucleation and growth near the CNTs.

The present work aims to explore and explain the crystallization behavior of PEEK–CNT nanocomposite feedstock filaments through the FFF process and institute a basis of fundamental knowledge that has not been previously investigated. This crystallization behavior is further correlated with mechanical and thermal properties of the printed parts, which expands on previously established systems. We will show that AM is aided synergistically via the addition of nanomaterials and affects the formation of crystalline and amorphous regions and thus governs the final properties. A series of nanocomposite filaments were fabricated via melt compounding with 1, 2, and 3 wt % (0.006535, 0.01314, and 0.019815 vol %, respectively) CNT contents and printed with the FFF process. Mechanical testing, thermal characterization methods, and both small- and wide-angle X-ray scattering (SAXS and WAXS) techniques were used to track and compare the formation of crystals following nanocomposite fabrication and the FFF process. MD simulations were employed to get insights into atomic-level mechanisms during the formation of organized PEEK chains around CNTs.

## 2 Materials and Methods

### 2.1 Materials and Equipment

Multiwall carbon nanotubes (NC7000, Nanocyl, Belgium), with an average diameter of 9.5 nm, an average length of 1.5 μm, and a density (ρ) of 2 g/cm^3^, were utilized in this study. The Arlon 1000 neat PEEK pellets were supplied by Greene Tweed (USA). A Process 11 twin screw extruder (Thermo Scientific) accompanied by a Thermo Scientific conveyor belt and a filament spooler manufactured by Filabot were used to extrude nanocomposite filaments with a diameter of approximately 1.75 mm to be used for FFF. The slicing software used to generate G Code for printing was Slic3r. The FFF 3D printer used was a Hyrel 16AS printer equipped with a 400 × 300 mm print bed with a 200 °C heating capability and an HT1-450 filament head with a 0.5 mm diameter nozzle and 450 °C maximum heating capability.

### 2.2 Nanocomposite Filament Manufacturing

**Figure 1:**
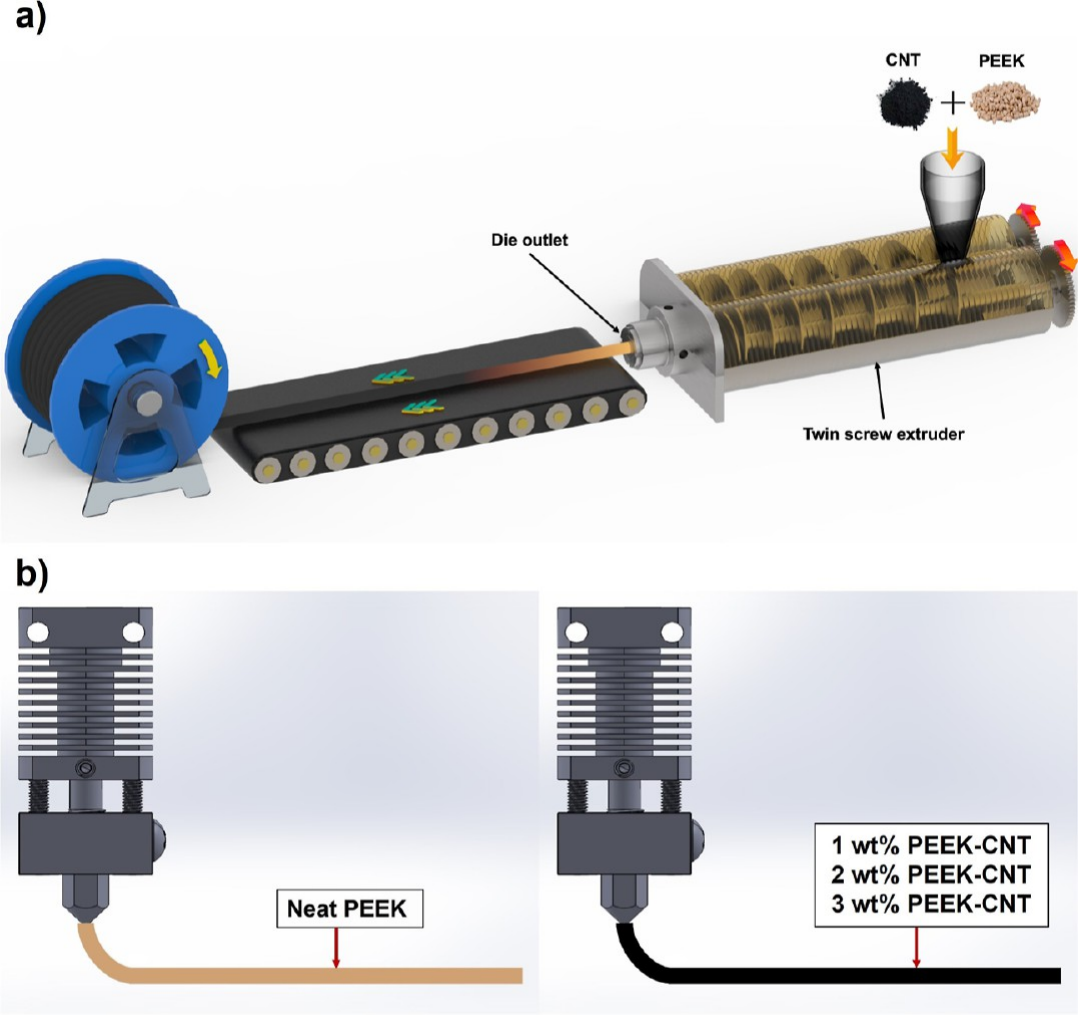
PEEK–CNT nanocomposite filament fabrication procedure. (a) Melt compounding of the PEEK pellet and pristine CNT via a twin screw extruder and an extrusion die into a 1.75 mm diameter filament. (b) FFF printing of each manufactured filament concentration including neat PEEK, 1 wt % PEEK–CNT, 2 wt % PEEK–CNT, and 3 wt % PEEK–CNT.

To create the filaments used for characterization, three concentrations of CNTs were selected and combined with PEEK: 1, 2, and 3 wt %. In addition to these three nanocomposite filaments, a neat PEEK filament was extruded and used as a control for all experimental work. The CNTs and PEEK were individually weighed and manually mixed prior to extrusion via a twin screw extruder. To avoid the use of harsh solvents, the twin screw extruder was selected as the optimal method for melt compounding of the polymer and nanoparticles into a homogeneous mixture. The twin screw extruder was heated to 375 °C and equipped with a 2 mm diameter circular die for filament extrusion. To manufacture the filament, the PEEK–CNT mixtures were deposited in the hopper of the extruder and met by a conveyor belt system at the die outlet and, ultimately, the filament spooler. This process is illustrated in Figure 1a,b. The diameter of the filament averaged 1.75 mm in diameter, and approximately 150 g of each concentration was extruded (neat PEEK, 1 wt % CNT, 2 wt % CNT, 3 wt % CNT.)

### 2.3 Printing Samples for Characterization

Samples were printed using a Hyrel Hydra 16AS system, equipped with a Hyrel HT1-450 printer head, and a GeckoTek Ez-Stik Hot build plate liner that offered more consistent adhesion for the first layer of the build. Each sample was printed using Slic3r’s rectilinear pattern, which has layers that alternate between longitudinal (0°) and transverse (90°) directions. The printing parameters used for each sample are offered below in Table 1.

**Table 1:** 3D Printing Parameters for Samples Manufactured with Hydra 16AS

3D printing parameters	
printing temperature	405 °C
print bed temperature	105 °C
print speed	20 mm/s
retraction speed	10 mm/s
cooling fan	off

### 2.4 Characterization Techniques

#### 2.4.1 Scanning Electron Microscopy

To study the interlayer behavior of short beam shear and flexural samples, a JCM-5000 NeoScope tabletop scanning electron microscope at a 10 kV acceleration voltage and high vacuum mode was used. To optimize the image quality, each of the samples evaluated were coated with gold for 60 s using a Cressington plasma sputter.

#### 2.4.2 Thermogravimetric Analysis and Specific Density

Thermogravimetric analysis (TGA) studies were performed using the TGA Q500 manufactured by TA Instruments. To study the CNT content of the nanocomposite filaments, each of the samples was heated from 30 to 1000 °C at a rate of 10 °C/min in a nitrogen atmosphere. An average measurement was achieved by using equal quantities of samples taken from the beginning, middle, and end of filament spools to account for any discrepancies in the concentration. The specific densities of the samples were measured using the water displacement technique outlined by ASTM D792. Each data point is the average of at least seven measurements.

#### 2.4.3 Differential Scanning Calorimetry

Differential scanning calorimetry (DSC) was performed using a TA Instruments Discovery DSC 2500. The procedure used began with a temperature ramp at a rate of 10 °C/min to 400 °C, was held at 400 °C for 5 min, and finally cooled at a rate of 10 °C/min to 40 °C. The samples evaluated in DSC tests were printed roads to account for the effect that the AM process has.

#### 2.4.4 Mechanical Testing

Tensile testing according to ASTM D638 was completed using an MTS Insight machine equipped with a 30 kN load cell. The test speed utilized was 5 mm/min, as dictated by the ASTM standard. Strain measurements were captured using direct image correlation (DIC) via Vic2D software. Each data point is the average of at least five samples.

To measure the interlayer shear strength (ILSS), short beam shear (SBS) testing (ASTM D2344) was used. The SBS testing was conducted using a Universal United STM system that was equipped with a 2 kN load cell and a three-point bending setup with a span of 1″ (∼25 mm) for all samples. The displacement rate and span-to-thickness ratio were set at 2 mm/min and 4, respectively. Each data point is the average of at least five samples. This property is currently highly sought after for parts created via AM, as it is notoriously difficult to achieve, causing parts to fall short of the out-of-plane strength required to be used in service.

Flexural properties of the nanocomposite samples were measured using a Universal United STM system equipped with a 2 kN load cell and a three-point bending setup, according to ASTM D790. The support span used was 2.4″ (∼61 mm), which equates to 16 times the thickness of the test specimen. The displacement rate and span-to-thickness ratio were set at 2 mm/min and 4, respectively. Each data point is the average of at least five samples.

#### 2.4.5 WAXS and SAXS of Filaments and Single Printed Roads

A Xenocs Xeuss 3.0 system was used for evaluating both filament feedstock and single printed roads for crystallinity and orientation of the CNTs and polymers. Measurements were performed under vacuum. Samples had an exposure time of 120 min, with the line eraser feature enabled for each measurement. All data analysis on orientation (Herman’s orientation parameter) and crystallinity was completed using XSACT 2.4 and plotted in Origin Pro. Note that samples were mounted at a negative 45-degree rotation angle.

### 2.5 MD Simulation Model and Technique

All-atom MD simulations were performed to investigate how the presence of aligned CNTs affects the dynamical and structural properties of chains in the printed nanocomposite. The simulations were performed using LAMMPS (large-scale atomic/molecular massively parallel simulator), an open-source package developed by Sandia National Laboratories.^34^ BIOVIA Materials Studio software was utilized for the initial setup of the molecular system.

The MD simulations provide valuable computational insights into the early stages of PEEK crystal nucleation near CNTs. The extent of change in the conformation, density, and energetics of PEEK chains is studied, which enables relating atomic-scale features to macro-scale properties of the system. The structural changes in the PEEK chains and the enhanced interaction are achieved as a contribution of both geometrical confinement and molecular interactions with CNTs. Due to the time-scale limitations of all-atom MD simulations, the simulations were performed under an isothermal ensemble and at the crystallization temperature.

**Figure 2:**
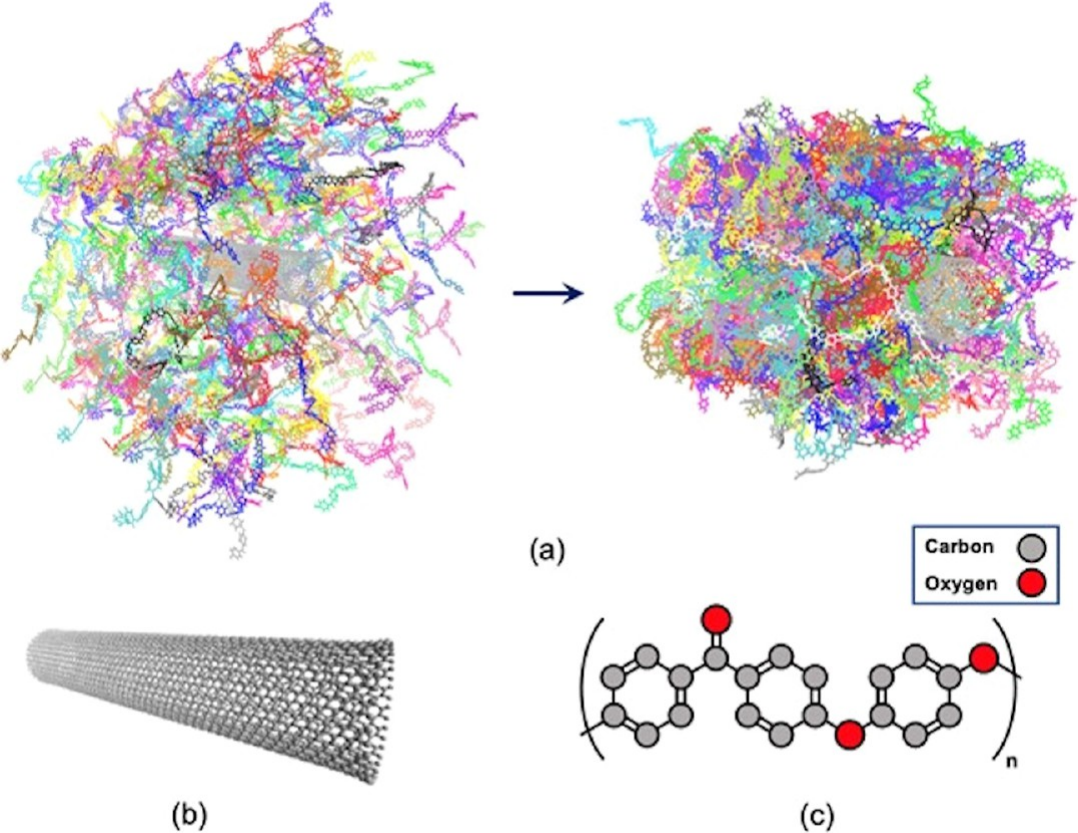
(a) Snapshots of 421 PEEK chains surrounding the CNT before (left) and after (right) the densification process. Each chain consists of five monomers and is denoted by a different color; (b) modeled single-walled carbon nanotube and (c) molecular structure of the PEEK monomer backbone.

The molecular structure of the PEEK monomer, with 34 atoms, and the single-walled carbon nanotube with 3672 atoms is shown in Figure 2a. Each modeled chain in this study includes five monomers to keep the contour length of the chains below the SWCNT length, 137 Å. To avoid the unsaturated boundary effect, the carbon atoms at the edges are capped with 38 hydrogen atoms. The SWCNT atoms were immobilized during the simulations by setting their forces and velocities to zero. The simulated SWCNT has a chirality of (20, 0). The cubic cell with periodic boundary conditions, Figure 2, consists of the SWCNT positioned in the center, surrounded by 421 polymer chains packed using the “Amorphous Cell” module in Materials Studio. This configuration results in an initial density of 0.098 g/cm^3^ and a total of 72,716 atoms. Care was taken in modeling the molecular system to ensure that the opposite parts of the SWCNT from two adjacent cells do not interact. Due to periodic boundary conditions, the response of PEEK chains is also influenced by the CNTs in the neighboring cells. In the resulting infinite system, the weight fraction of CNT is 7.3%. It is noted that a big enough CNT was modeled to make conforming of PEEK chains possible, and the CNT weight fraction of the infinite system will not affect the desired outcomes of the current simulation close to the CNT surface.

The system pressure and temperature were controlled at the required level using a Berendsen barostat and a Nosé–Hoover thermostat, respectively.^35,36^ This barostat has been shown to give similar system density fluctuations as a more developed Parrinello–Rahman barostat, leading to proper local polymer nanostructures. An extended version of the polymer consistent forcefield (PCFF), named PCFF+ and developed by MedeA commercial molecular simulation software, was used to define intra-molecular interactions.^37,38^ Partial charges were also calculated by the forcefield assign option of this software. The intermolecular interactions were simulated by electrostatic interaction energy and van der Waals (vdW) terms in the PCFF forcefield. Long-range Coulombic interactions were treated using a particle–particle particle-mesh (pppm) solver, which is a more efficient choice for big systems compared to the conventional Ewald method.^39^ Lennard-Jones interactions were truncated at the cut-off radius of 20 Å. The employed Lennard–Jones (class 2 form) potential in this study has the form
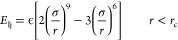
1where *r*_c_ is the cutoff radius. After energy minimization, the simulation box with initial dimensions of 200 × 200 × 300 Å^3^ was equilibrated at high pressure (800 atm) and temperature (750 K) in an NPT ensemble for 100 ps with 0.5 fs time steps, during which voids are removed. Besides treating the voids, a high initial pressure brings the chains in at a faster pace and decreases chain folds raised by the low initial density of the system before building denser chain clusters. Subsequently, the system pressure was turned down through three increments in the *NPT* ensemble, each 100 ps. The neat PEEK was densified following the same procedure. Smooth density profiles after the pre-equilibration stage indicated a proper distribution of the chains and a well-equilibrated system. Following the pre-equilibration stage, a long equilibrium run, from which the main data sampling has been conducted, was carried out for 150 ns with 1 fs time steps. The pressure and temperature in the long run were held constant at 1 atm and 500 K, respectively.

An important aspect of studying semi-crystalline systems having CNTs is the promoted crystallization due to the large surface area that CNT offers for the polymer chains, which act as possible nucleation sites. CNTs have been shown to induce crystallinity even in amorphous polymers that do not crystallize under normal conditions.^40^ In the current study, we intend to simulate the pre-crystallization stage right after polymer chains come into contact with the CNT and the subsequent ordering.

## 3 Results and Discussion

### 3.1 TGA and Specific Density

The TGA testing completed on all PEEK–CNT filament concentrations revealed the experimental CNT content in the nanocomposite, which can be compared to the estimated content during manufacturing, shown in Table 2. The lowered concentration can be attributed to the loss of CNTs during the filament extrusion process as well as differences in the concentration throughout the 150 g of filament itself due to potential agglomerations.

**Table 2:** Actual Content of CNTs in PEEK–CNT Nanocomposite Filaments as Determined from TGA Testing

sample	experimental CNT concentration from TGA (wt %)
1 wt % CNT	0.64
2 wt % CNT	1.80
3 wt % CNT	2.31

Overall, the thermal degradation behavior of the PEEK–CNT filaments is nearly identical to that of the neat PEEK filaments, as displayed in Figure S1a. The steep fall on the curve, beginning around 560 °C, corresponds to the beginning stages of degradation of the PEEK polymer itself, although it only degraded to approximately 51–53%, according to the concentration of CNTs present in the material. Comparatively, the CNTs themselves did not degrade significantly when heated to 1000 °C, implying that their thermal degradation and weight change are negligible. It is concluded that the addition of the CNTs did not alter the thermal stability of the matrix, which is imperative for the potential aerospace applications that are implied with this material.

The density for all the samples is listed in Table 3, and the increased concentration of CNTs has a direct correlation with the decreased density in the samples. This is attributed to increasing the concentration of the nanotubes, which causes restriction of flow in the heated nozzle during the deposition of individual roads and results in voids throughout the printed part. As shown in the SEM images in Section 3.7, the voids occur more frequently at higher concentrations of CNTs and subsequently contribute to lower interlayer shear strength and poor adhesion between printed roads. The slight increasing trend among the nanocomposite samples, however, is a result of the higher density of the CNTs themselves, as modeled by the idealized density calculated using the rule of mixtures.

**Table 3:** Calculated and Experimentally Derived Specific Density Measurements of PEEK–CNT Nanocomposite Samples, Exhibiting a Decreasing Trend as the Concentration of CNTs Increases due to the Influx of Voids throughout the Printed Part

sample	calculated density (rule of mixtures) [g/cm^3^]	specific density [g/cm^3^]
neat PEEK	1.3	1.056 ± 0.036
1 wt % CNT	1.307	0.726 ± 0.057
2 wt % CNT	1.314	0.753 ± 0.066
3 wt % CNT	1.321	0.808 ± 0.032

### 3.2 Differential Scanning Calorimetry

**Figure 3:**
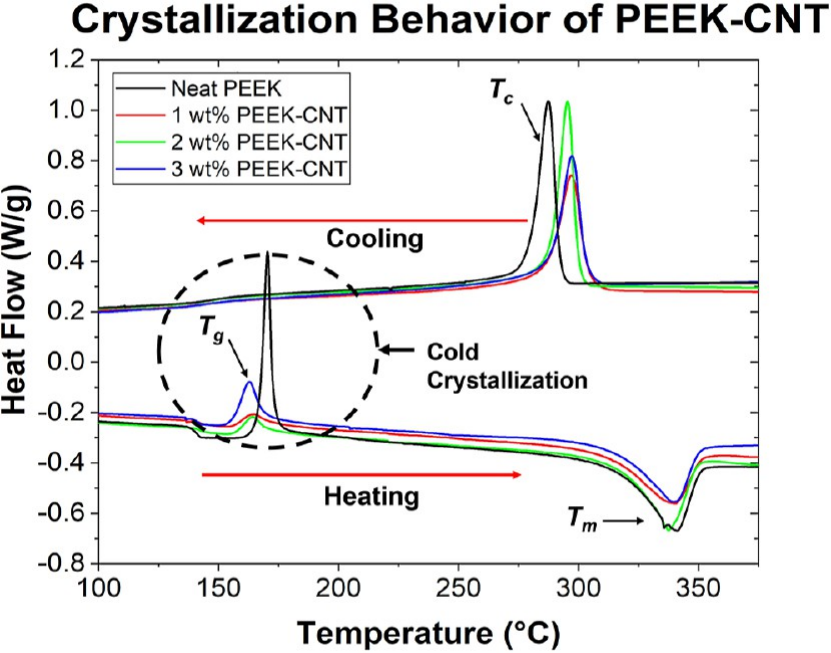
DSC of PEEK–CNT printed roads, displaying the glass-transition temperature (*T*_g_) and melting temperature (*T*_m_) on the heating curve and crystallization temperature (*T*_c_) on the cooling curve. Cold crystallization is exhibited at *T*_g_ during heating, where amorphous regions of the polymer reassemble into crystallites before ultimately melting at *T*_m_. An increase in crystallization temperature (*T*_c_) is observed in CNT nanocomposite concentrations, which allows crystallite formation in rapid-cooling environments.

The crystallization behavior of PEEK with added CNTs was studied through means of DSC experiments on extruded feedstock material, and the results are summarized in Figure 3 and Table 4. The neat PEEK and nanocomposite concentrations experience the same thermal transitions, which are the glass-transition temperature (*T*_g_), the melt temperature (*T*_m_), and the crystallization temperature (*T*_c_). During the endothermic heating process, the first thermal transition (the *T*_g_) occurs at approximately 165 °C and the second thermal transition (the *T*_m_) occurs at 340 °C. During the cooling process, the final thermal transition (the *T*_c_) occurs near 290 °C, where crystallites begin to nucleate and make up the semi-crystalline microstructure.

**Table 4:** DSC Melting and Crystallization Temperatures and Enthalpies, along with Crystallinity of PEEK–CNT Samples

	cold crystallization temperature (°C)	*T*_m_ (°C)	*T*_c_ (°C)	Δ*H*_m_ (J/g)	Δ*H*_C_ (J/g)	*X*_c_
neat PEEK	170.16	340.72	287.43	34.14	40.26	30.97
1 wt % PEEK–CNT	162.54	337.22	295.44	31.80	40.21	30.92
2 wt % PEEK–CNT	163.81	339.10	297.42	28.73	33.98	26.14
3 wt % PEEK–CNT	165.81	339.91	297.13	34.11	34.67	26.67

The most notable result that is derived from the testing is the non-isothermal cold crystallization that occurs during heating, shortly after the glass-transition temperature is achieved, ranging approximately between 162 and 170 °C, as shown in Table 4. The cold crystallization phenomenon occurs when the trapped amorphous regions of a semi-crystalline polymer are heated above the *T*_g_ and allow the polymer chains to achieve enough mobility to create a crystal structure for a short time, before the melt temperature is reached. Although this phenomenon has not been extensively studied, it has been observed by a few researchers who have experienced similar DSC curves in polymers such as poly (aryl ether ketone) and poly (lactic acid).^41,42^ It is hypothesized that the AM of these nanocomposites causes the cold crystallization to occur, attributed to the rapid cooling that the polymer experiences after it is extruded from the nozzle and onto the print bed or adjacent layer. The rapid cooling environment is conducive to immobilizing amorphous regions of PEEK before the polymer chains have the chance to form crystalline regions. PEEK–CNT concentrations exhibit less cold crystallization than neat PEEK because the CNTs initiate the polymer crystallization more rapidly due to their nucleating effects and the increased crystallization temperature. Additionally, there is a confinement effect on the polymer chains in the presence of the CNTs, which restrict movement and contribute to the lower quantities of cold crystallization experienced in the material. Cold crystallization only occurs in the presence of a rapidly cooled environment, and it does not provide the necessary time for the polymer chains to organize into crystallites. Therefore, when the cooling rate is lower and more controlled, as seen in the cooling curve displayed in the DSC analysis; the cold crystallization phenomenon ceases to appear.

**Figure 4:**
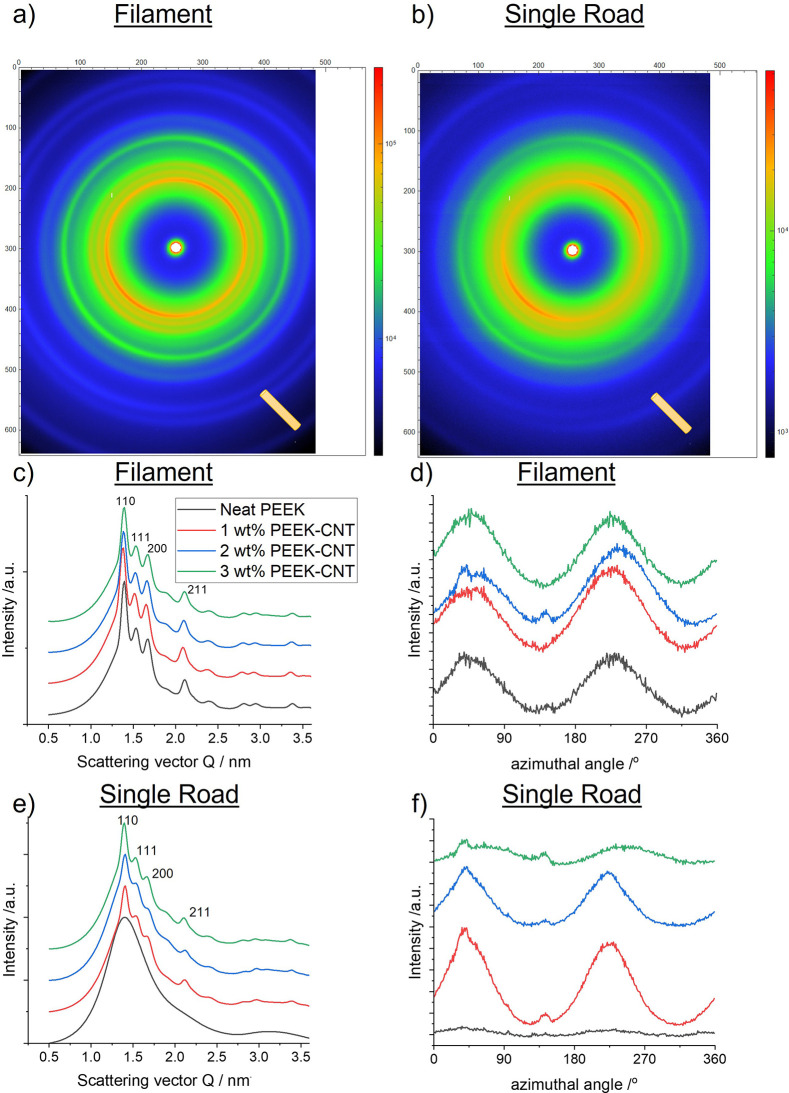
Wide angle X-ray scattering data for filaments and roads. (a) Filaments and (b) roads 2D WAXS patterns that show partial orientation of the polymer (arcs). The orientation of the filament and road is negative 45° (indicated by the bottom right road). (c,e) Diffraction patterns showing crystalline phase of PEEK with typical 110, 111, 200 and 211 peaks. (d,f) Azimuthal scans through the 110-diffraction peak showing the degree of orientation of the polymer.

**Figure 5:**
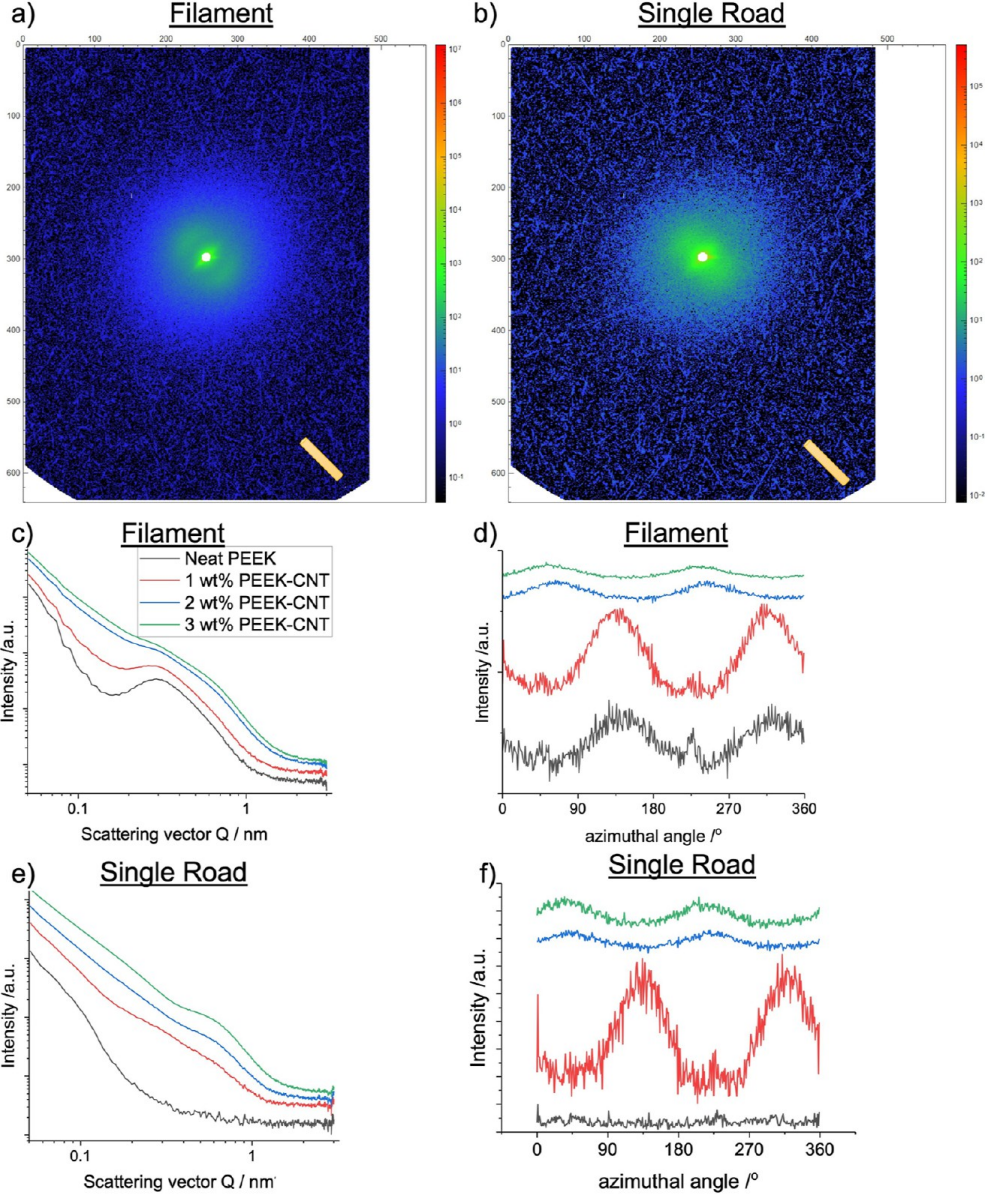
Small-angle X-ray scattering data for filaments and roads. (a,b) 2D patterns with anisotropic low *Q* intensity distribution and orthogonal alignment of CNTs and PEEK crystal long period. (c,e) Averaged intensity distribution with *Q* and the influence of CNTs and printing on crystal formation. (d,f) Azimuthal intensity distribution at *Q* = 0.25 Å^–1^.

**Figure 6:**
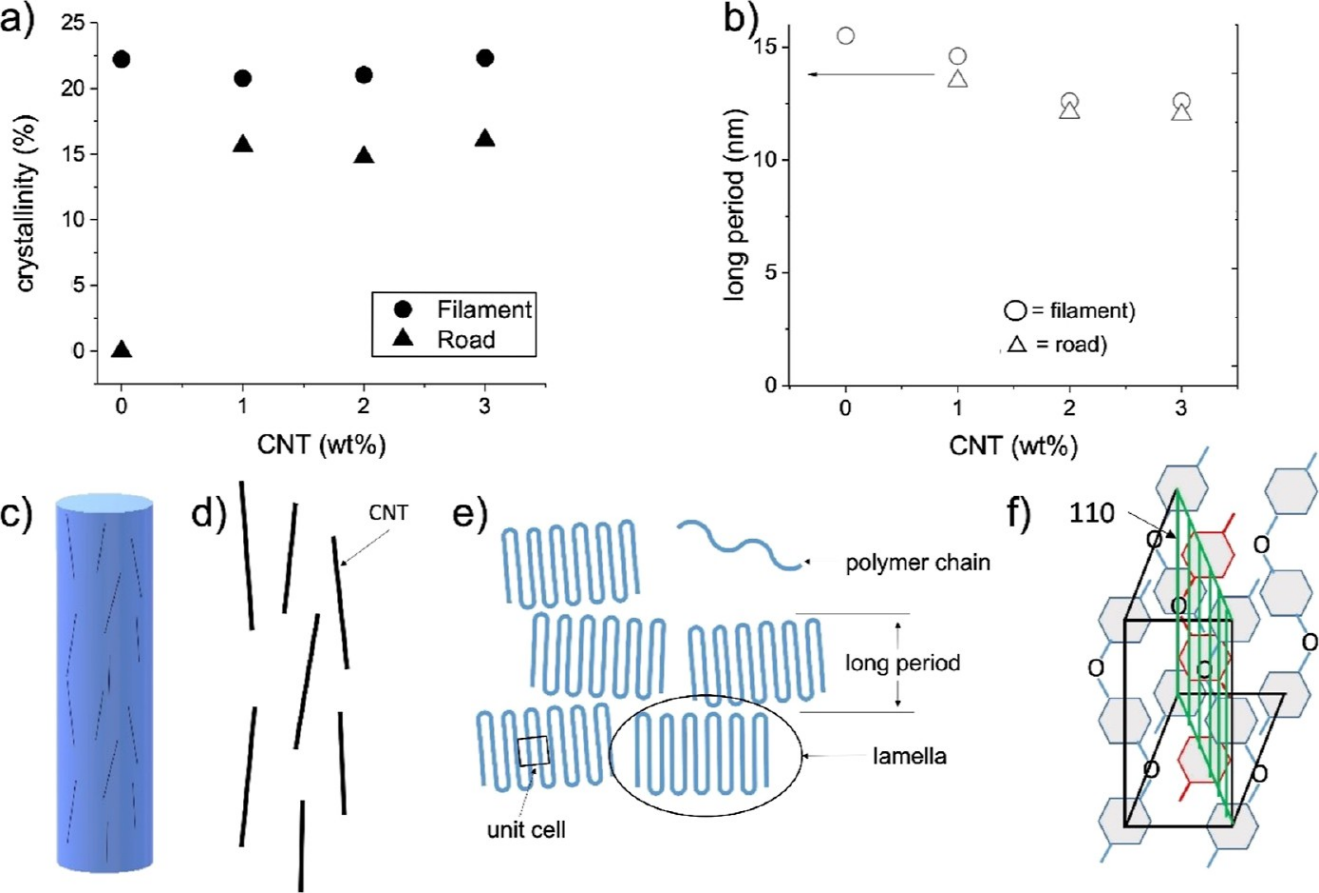
Crystallinity (fraction of 1) and long period dimensions from wide-angle X-ray and small-angle X-ray scattering. (a) Crystallinity calculated from the ratio of total intensity of crystalline and amorphous peaks. (b) Long period dimension calculated from integrated intensity and peak position of the small-angle scattering peak. (c) Single road with aligned carbon nanotubes along the print direction. (d) Alignment of carbon nanotubes along the printing direction and (e) aligned morphology of polymer with respect to (e). The polymer forms lamellae during crystallization, and lamellar stacks are formed. The distance between the stacks is called the “long period” for semi-crystalline polymers. (f) Polymer crystallizes into an orthorhombic unit cell. The 110 plane is along the length of the polymer chains and along the printing direction.

In addition to the cold crystallization, the crystallization temperature during cooling for all PEEK–CNT concentrations was raised by values ranging from 8 to 10 °C when compared to the neat polymer, as seen in Figure 3. The increase in *T*_c_ implies that the added CNTs cause the crystallization to occur earlier in the cooling process than that of neat PEEK (Figure 3) and lead to more crystallites within the nanocomposite due to increased polymer chain mobility at these temperatures. This is an important processing factor in FFF printing because it allows for crystallization in a rapid cooling environment, while the neat polymer was shown to have more amorphous regions, as seen in the cold crystallization on the heating curve above in Figure 3 and discussed further in the X-ray scattering section, Figures 4–6. The increased crystallization temperature due to CNT addition can be translated into other manufacturing methods such as injection molding, where rapid crystallization can be difficult to achieve.

The crystallinity level, expressed as *X*_*c*_, was calculated by using 130 J/g as the extrapolated value of enthalpy according to the melting of a 100% crystalline sample for Δ*H*_m__,PEEK_.^43^ Although the CNTs were seen to increase crystallite nucleation behavior in the PEEK matrix in other studies, the rapid cooling environment experienced during printing is not present in DSC scans here, which can impact the crystallinity level.^16,28^ Lower crystallinity was seen in the 2 and 3 wt % nanocomposite concentrations and can be attributed to the spatial confinements by the CNT nanofillers as well as the controlled cooling rate. Considering a hypothetically dispersed system, the estimated distance between CNTs in the 1, 2, and 3 wt % concentrations is 336, 235, and 190 nm, respectively, which corresponds with the confinement effect present in the increasing nanoparticle concentrations. This trend is also attributed to the opposing effect that CNTs have in the system. The CNT inclusion increases crystallite nucleation, which is observed in the 1 wt % concentration, while simultaneously confining the movement of the polymer chains. Increasing the CNT concentration provides additional nucleation sites, increasingly prohibits chain movement, and will begin to exhibit decreased crystallinity, as observed most notably in the higher CNT concentrations. This is explained further in succeeding sections.

### 3.3 WAXS and SAXS of Filaments and Printed Roads

To evaluate the effects of processing on extruded filaments and printed roads, samples were characterized via X-ray diffraction. Figure 4 shows typical 2D WAXS patterns for filaments and roads (Figure 4a,b) and the reduced 1D data for each of the weight fractions (Figure 4c,e).

The 2D patterns for all weight fractions (Figure 4c–f) reveal an alignment in the intensity distribution that is consistent with flow or deformation-induced alignment of semicrystalline polymers.^44^ This is evidenced by the peaks that correspond with the typical 110, 111, 200, and 211 peaks of the PEEK unit cell for both extruded filaments and printed roads, as there is shear-induced alignment in both filament extrusion and material deposition processes. The strongest peak is from the 110 plane within the unit cell (Figure 4c,e) and is aligned along the printing direction. Overall, the polymer chains are also aligned along the printing and extrusion directions (Figure 4d,f), as evidenced by the azimuthal scans through the 110 plane, which had the highest intensity peak. The quality of orientation is not significant, and a Herman’s orientation parameter of 0.25 was calculated for both polymer alignment and CNT alignment. This can be seen in the broad intensity distribution in Figure 4c,e. Sufficient dwell time of the extruded filament in air allows the material to crystallize to similar overall crystallinity. On the other hand, the material is super-cooled during 3D printing when the 400 °C melt is deposited onto the 105 °C build platform, which is the limit of the used printer. The neat material is therefore amorphous. The use of more optimal printing conditions leads to partial crystallinity of printed PEEK.^10^ In this case, CNTs act as nucleation sites and despite the super-cooling effect, the 3D printed nanocomposite samples crystallized with an overall lower crystallinity compared to the extruded filament. As previously discussed, the lower crystallinity within the nanocomposite printed roads is attributed to the polymer confinement effect, where an increase in CNTs restricts polymer chain motion and, consequently, results in a lower overall crystallinity.

The SAXS data summarized in Figure 5 confirms the WAXS data and reveals additional effects of CNT addition on the crystallization of the PEEK. Figure 5a,b shows the alignment of the polymer, seen in the lobes of higher intensity along the direction of the extrusion and printing. The crystallinity data obtained via X-ray scattering is summarized in Figure 6a. The crystallinity between extruded neat and CNT-loaded filament is the same within error (Figure 6a). The SAXS also confirms the amorphous phase of the neat 3D printed PEEK and the overall lower crystallinity for the 3D printed samples compared to extruded filaments (Figure 6b). Both WAXS (Figure 4f) and SAXS (Figure 5f) show better alignment of the 3D printed 1 wt % CNT nanocomposite. We hypothesize that the confinement effects at these CNT volume fractions are optimal for CNT alignment.

The alignment is affected by the flow dynamics and crystallization of polymers in confined environments. Furthermore, the increase in CNTs also influences the size of crystallites, as seen in the calculation of the long period of PEEK (Figure 6b). With increasing CNTs, the size of the crystals decreases (Figure 6b). This is based on the decrease seen in the long period because of the increasing CNT concentration, where the size of the crystallites themselves is decreasing, due to the smaller space between lamellae. Figure 6c–e illustrates the alignment of CNTs and polymer crystals (lamellae) along the printing direction and defines the long period.^45^ The order between lamellae (Figure 6e) leads to meridional scattering peaks in SAXS, while the chain packing within the lamellae causes equatorial peaks (preferential alignment) in WAXS. The alignment of the CNTs is seen in the anisotropic intensity distribution very close to the beam stop (*Q* < 0.1 Å^–1^) and can be seen in Figure 5c,e. In these two cases, the scattering intensity of CNTs superimposes any intensity of the polymer. It is also obvious that the CNT alignment is orthogonal (equatorial) to the long period peak (meridional). The quality of orientation based on the intensity distribution is similarly weak as the polymer alignment.

### 3.4 Tensile Properties

**Figure 7:**
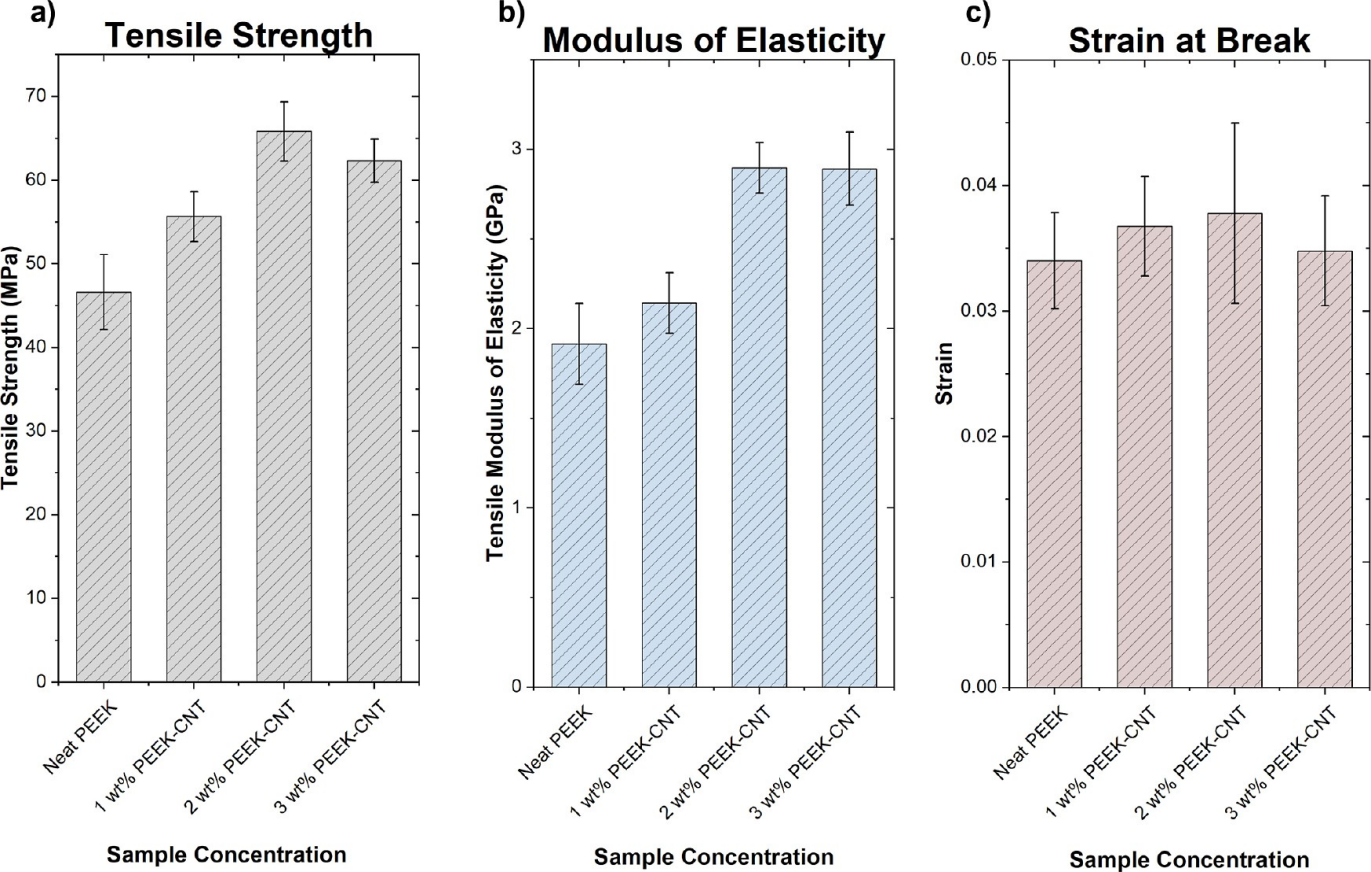
Tensile properties of PEEK–CNT composites including the (a) tensile strength, (b) modulus of elasticity, and (c) strain at break, which each increase due to the addition of CNTs which facilitates homogeneous nucleation while simultaneously confining polymer chain mobility, creating an opposing effect.

The addition of CNTs was expected to increase the tensile properties overall, due to increased crystallinity compared to the neat PEEK, as well as the reinforcement strength contributed by the CNTs and is shown in Figure 7. Modestly increased values for ultimate tensile strength are observed in all three nanocomposite concentrations. In fact, the tensile strength of the 1, 2, and 3 wt % nanocomposite samples increased by 21, 42, and 35%, respectively. Although there is still a slight increase in strength in the 2 wt % concentration, it is theorized that this concentration of CNTs begins to exhibit the early signs of the conflicting heterogeneous nucleation of crystallites and confinement of polymer chain mobility simultaneously. The restricted chain movement consequently lowers the number of crystallites that are nucleated within the polymer; however, the crystallites are hypothesized to be larger in size, which results in a smaller increase in tensile strength and eventually a drop at high CNT loadings. Additionally, the SAXS and WAXS data shows the alignment of crystallites and CNTs, which further increases the tensile strength as the polymer chains and CNTs are oriented in the direction of the applied tensile stress. With the elastic modulus, there is an increase in the material stiffness in 1, 2, and 3 wt % concentrations by quantities of 12, 51, and 39%, respectively. As seen with the tensile strength, the contributions for the increased elastic modulus values are due to the stiffening effects from the CNTs themselves and the crystallites nucleated on the surface of the CNTs. The values for strain at break are similar throughout the samples, with the neat PEEK sample exhibiting a similar result because it is largely amorphous, giving it better toughness than crystalline regions.

### 3.5 Interlayer Shear Strength

**Figure 8:**
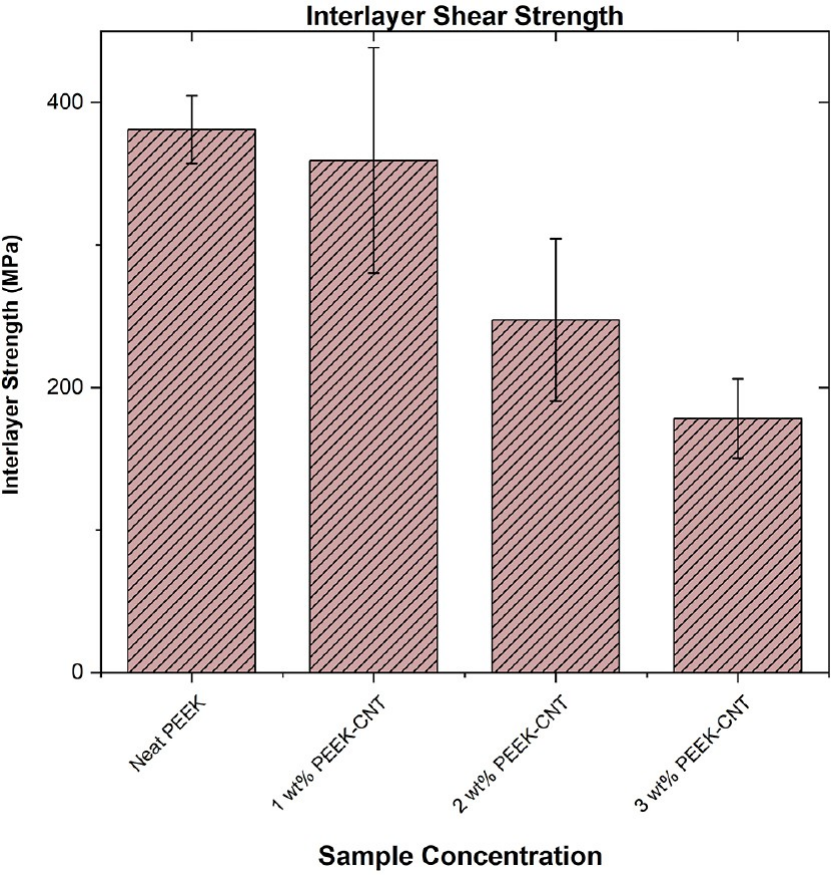
Interlayer shear strength determined by short beam shear testing of PEEK–CNT composites, with decreasing interlayer adhesion due to restricted polymer chain mobility causing low interdiffusion between printed roads and a high void content.

The interlayer shear strength (ILSS), determined through short beam shear testing, reports the adhesion between the layers through means of stress applied in a three-point bend mode. Interlayer adhesion occurs when adjacent printed roads are welded or bonded together through molecular interdiffusion and entanglement of polymer chains across the joining interface. In this case, the interlayer shear strength in the PEEK–CNT nanocomposite samples exhibits a decreasing trend that is a result of poor interdiffusion between printed roads and the preceding layer, as shown below in Figure 8. As described in the preceding morphology discussions, one of the main causations for the reduced ILSS is that the aligned CNTs produce a barrier effect on the diffusion of PEEK polymer chains at interfaces. This effect limits the mobility of the chains, specifically hindering their ability to diffuse between roads and adjacent layers, creating a weak bond that is unable to resist shear stress as well as the neat polymer. Increasing the concentration of CNTs further exaggerates this effect, which is seen with the 3 wt % CNT sample, where the ILSS is much lower than its neat, 1, and 2 wt % nanocomposite counterparts. Generally, an increase in crystallinity results in improved interlayer adhesion, and with a decreasing trend observed in the crystallinity of the printed roads, this contributes to the lower interlayer strength.^46^ Even though the PEEK polymer that is extruded from the nozzle, at approximately 400 °C, is significantly above the *T*_g_, would typically allow for increased movement of polymer chains; this is not the case with CNT addition. The limited chain mobility because of CNT addition produces a weak interlayer bond, despite heating the material above its glass-transition temperature. Additionally, the extreme thermal gradient that is present in the printed samples induces a residual stress throughout the part, which results in warpage, or the tendency of layers to pull away from the print bed and adjacent printed surfaces. The lower interlayer shear strength is also attributed to the presence of voids that occur during printing. The CNT additions restrict the flow of the melt from the print head, with higher concentrations of the nanoparticles causing the appearance of voids. This, coupled with the polymer chain confinement causing limited diffusion between the layers, ultimately contributes to the diminished interlayer shear strength. An increased volume of voids further lowers the interlayer adhesion and produces a tendency for delamination and low out-of-plane strength for these nanocomposite parts. It is hypothesized that the ILSS can be enhanced through means of heat treatments, such as post-process annealing.^46^ However, these post-processing techniques were not the focus of this study.

### 3.6 Flexural Properties

**Figure 9:**
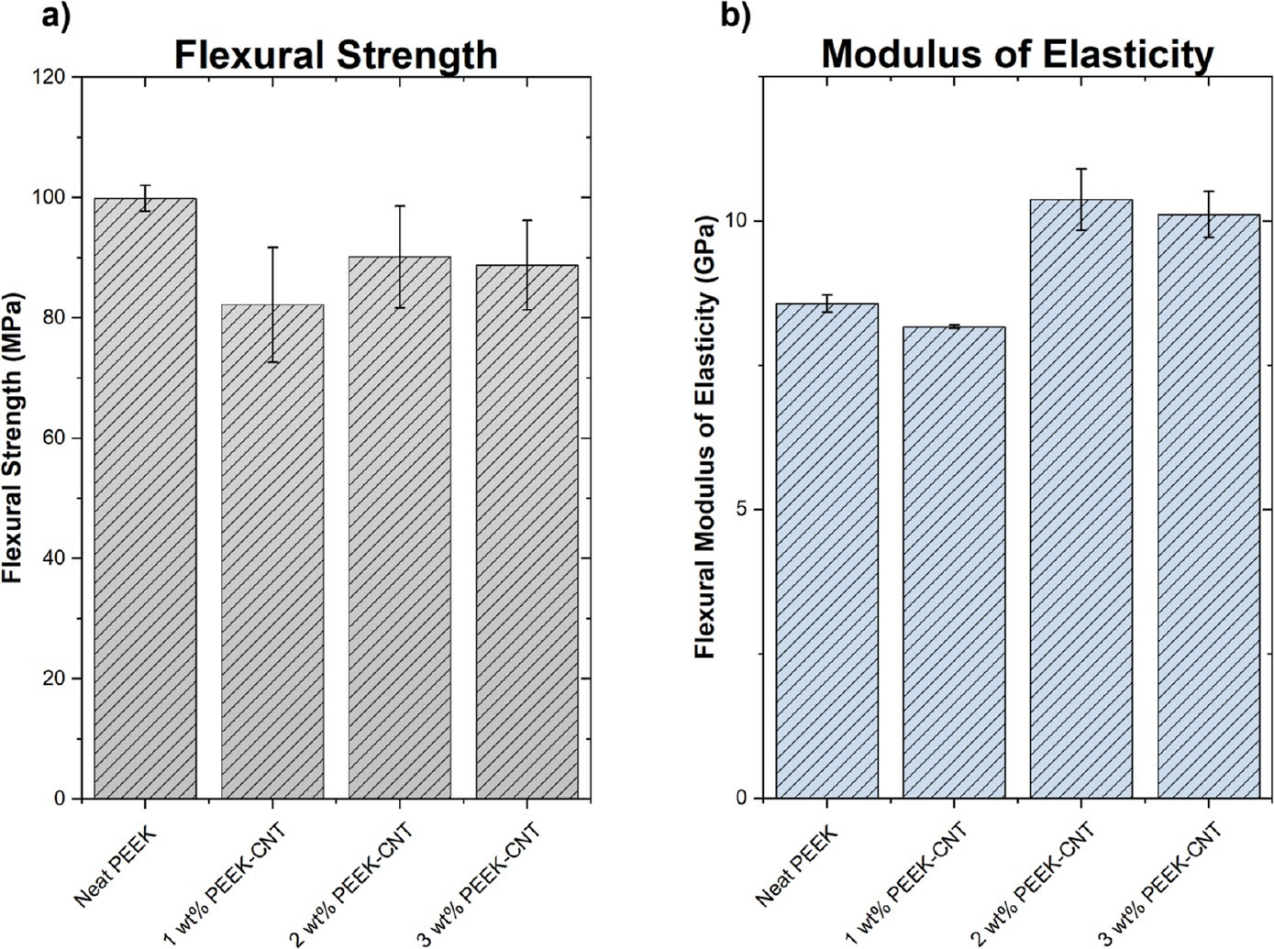
Flexural properties of PEEK–CNT composites including the (a) flexural strength and (b) flexural modulus of elasticity with increased values for the elastic modulus attributed to increased crystallinity and directionality.

The flexural properties of PEEK–CNT are displayed in Figure 9. There is a slight decrease in flexural strength of all three nanocomposite samples when compared to neat PEEK, which signals that the addition of CNTs into the PEEK matrix does not affect this property significantly, and the material is able to exhibit similar properties to neat PEEK itself (see Figure 9a). Unlike the interlayer shear strength determined through short beam testing, the flexural strength samples have a smaller thickness. This means that these parts are closer to the heated print bed and experience a smaller temperature difference throughout the sample during printing. The decreased thermal gradient results in less residual stress due to warping in the samples, and therefore, there is less tendency for layers to delaminate. Furthermore, the flexural strength is dependent on the interlayer strength to a degree, which is why there is not a significant increase in strength shown in the samples, as was the case with the tensile samples that experienced stress applied parallel to the interlayer plane. These two phenomena act as an opposing effect in flexural strength, where the tensile strength showcases an increase, while the interlayer shear strength models a decreasing trend, and the resulting strength is not largely dependent on the CNT addition. Therefore, if the thermal gradient is minimized during manufacturing, there is a lower likelihood for the printed parts to delaminate and ultimately fail. The modulus of elasticity in this flexural mode, however, does increase in the 2 and 3 wt % PEEK–CNT concentrations of the nanocomposite from the addition of the nanoparticles to the PEEK matrix due to the high stiffness of CNTs and an increase in crystalline regions that nucleate on the surface of the nanotubes themselves (see Figure 9b). As discussed previously with the tensile properties, it is hypothesized that the alignment of the crystallites within the printed road contributes to an increase in the material’s stiffness and resistance to deformation from applied stress. In this case, the flexural Young’s modulus value of the three PEEK–CNT concentrations are nearly the same, which suggests that the CNT content itself is not responsible for the increased elastic modulus.

#### 3.6.1 Scanning Electron Microscopy

**Figure 10:**
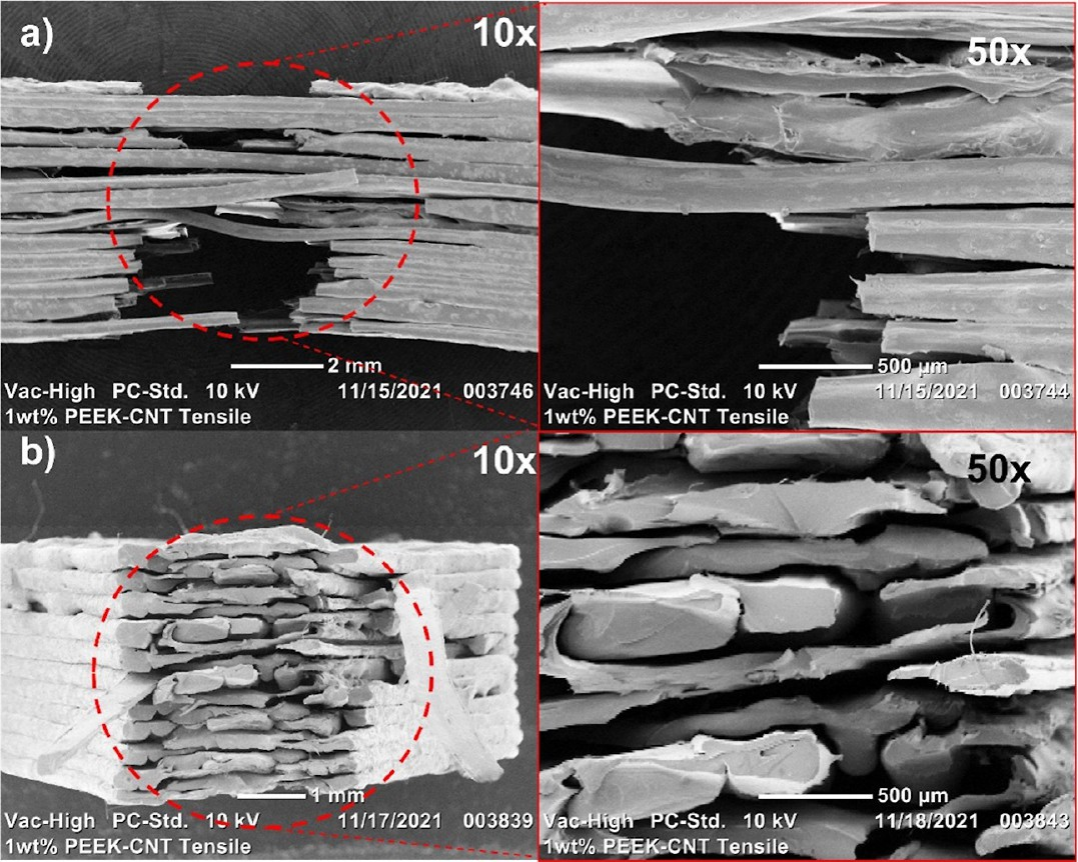
SEM images of the fracture behavior of the 1 wt % PEEK–CNT tensile sample (a) normal to the fracture surface and (b) fracture surface. The material exhibits behavior characteristic of traditional composite materials, with layers delaminating and fracturing at different points.

SEM imaging was used on short beam shear (SBS) samples and 1 wt % PEEK–CNT tensile samples to evaluate both the delamination and fracture behavior observed in their respective characterization tests. In Figure 10, the SEM images of the 1 wt % CNT tensile samples show the fracture behavior from a side profile (Figure 10a) as well as the surface that is normal to the tensile force being applied to the specimen (Figure 10b). The non-linear fracture shown in Figure 10 is characteristic of traditional composite materials, where the transfer of stress from the PEEK matrix to the CNT reinforcement allows for individual layers to fracture without causing ultimate failure of the part. This allows the nanocomposite material to withstand larger forces than the neat PEEK control sample by evenly redistributing the force applied among the CNTs. Furthermore, with the FFF process itself creating parts via a layer-by-layer process composed of single printed roads, the individual roads within the printed part enable the ability to fracture independently of their neighboring roads and of adjacent layers, which allows for a higher tensile strength to be achieved. The ultimate failure of the sample will not occur due to a singularly printed road or even printed layer, but rather once several of the layers start to experience failure, as seen in Figure 10a.

When evaluating the fracture behavior of the surface normal to the tensile force applied to the sample, each of the individual roads is visible on the surface (see Figure 10b). Notably, the individual roads each experience what appears to be a brittle failure due to the addition of CNTs into the PEEK matrix. This is further confirmed by the increased tensile modulus of elasticity, or material stiffness, that was discussed in Section 3.4. Despite the low ductility in the material, the use of FFF to manufacture the sample aided in the increase of tensile strength, with each road acting as an individual strand rather than a bulk material as seen in traditional injection molding processes. In addition to the brittle fracture, Figure 10b exhibits some patterns of crazing where the PEEK matrix begins to experience small voids that will ultimately add to its failure mechanism.

**Figure 11:**
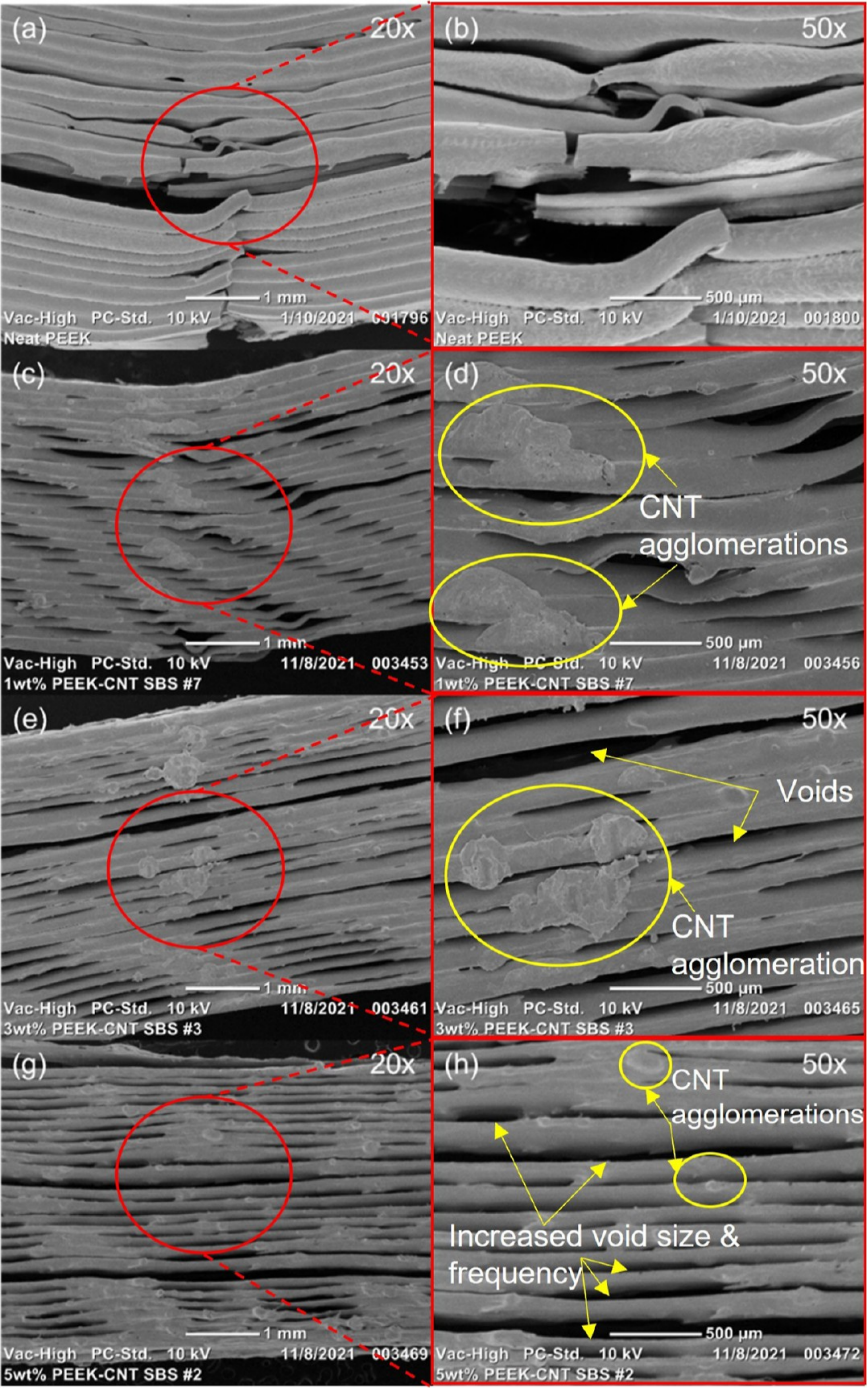
SEM images of short beam shear samples: (a) neat PEEK at 20× magnification, (b) neat PEEK at 50× magnification, (c) 1 wt % PEEK–CNT at 20× magnification, (d) 1 wt % PEEK–CNT at 50× magnification, (e) 2 wt % PEEK–CNT at 20× magnification, (f) 2 wt % PEEK–CNT at 50× magnification, (g) 3 wt % PEEK–CNT at 20× magnification, and (h) 3 wt % PEEK–CNT at 50× magnification.

Figure 11 shows the short beam shear samples following their three-point bending for evaluation of the interlayer shear strength. The nanocomposite samples exhibit lower interlayer adhesion than their neat PEEK counterparts, as seen in Figure 11 and as described in Section 3.5 previously. Despite the increased crystallinity in the nanocomposite samples, the inhibited diffusion of the polymer chains and visible porosity and voids in specimens led to the lower interlayer strength of PEEK–CNT samples.

The pattern displayed on the sides of the samples can be attributed to small CNT agglomerations that cause an inconsistent flow of nanocomposite material through the nozzle of the printer, resulting in periodic and repeating voids in the printed parts. As the concentration of the CNTs increases, the viscosity of the nanocomposite melt increases and results in a reduced flow rate, despite correctional efforts on the print parameters directly. As previously discussed, the 2 wt % concentration (Figure 11e) marks the beginning of the conflicting polymer chain confinement to the crystallite nucleation effect imparted by CNTs, which manifests in a reduced flow of the polymer itself. This is further exaggerated in the 3 wt % concentration (Figure 11g), where the printed roads appear smaller in diameter and feature larger voids between each point of interlayer adhesion. Here, the voids that are present in the sample are increased in both size and frequency throughout each layer. The void content was estimated using SEM images of the printed nanocomposite samples and was found to increase as the CNT concentration increased. Neat PEEK exhibited 0.39%, 1 wt % PEEK–CNT exhibited 9.88%, 2 wt % exhibited 10.97%, and 3 wt % PEEK–CNT exhibited 16.48% voids. Therefore, the lower flowrate has a negative impact on the interlayer adhesion of these printed parts. The CNT clusters themselves can be seen clearly in the 1 and 2 wt % samples (see Figure 11d,f) and are detrimental to the interlayer adhesion as well. With an increase in the CNT concentration, the formation of these agglomerates goes up in probability, which decreases the interlayer shear strength property in addition to the polymer chain confinement effects imparted by the CNTs. To improve the interlayer adhesion further, the viscosity of the nanocomposite melt would need to be altered to match the level of the neat PEEK to achieve the consistent flow rate that would prevent air pockets and voids from occurring. This could perhaps be achieved via a heated chamber during the FFF process, which would aid in lowering the thermal gradient between the heated bed and adjacent printed layers.

### 3.7 MD Simulation Results

**Figure 12:**
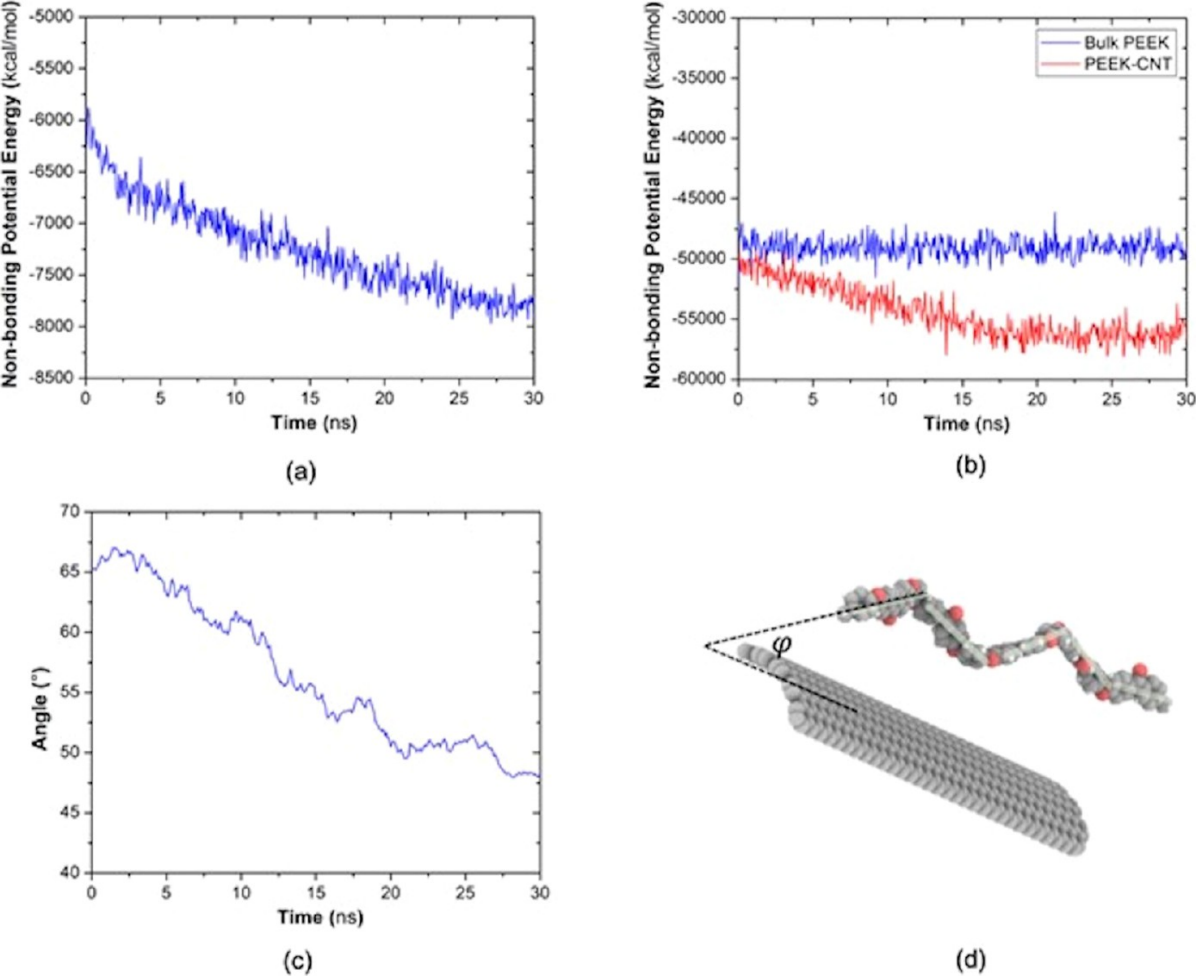
(a) Time evolution of non-bonding potential energy contribution between PEEK and CNT during the major ordering period. (b) Non-bonding potential energy between PEEK chains over time. (c) Mean angle of chain fragments with respect to the CNT axis in the recorded snapshots. (d) Schematic representation of defined fragment vectors on a PEEK chain and the angle between the CNT axis and the fragment vector.

The energetics of the system were studied based on the corresponding atomistic trajectories to understand the effect of CNTs on the intermolecular interactions between PEEK chains and between PEEK and CNT, as well as their relevance to the chain configuration. As no changes in the bonded interactions are present during the simulation, the change in the total potential energy is expected to be due to the changes in the non-bonding terms, including van der Waals (*E*_vdW_) and electrostatic (*E*_elec_) energies. The interaction energy between PEEK and CNT after equilibration can be calculated through considering the difference between the potential energy of the nanocomposite and the isolated components
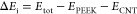
2where *E*_tot_ is the potential energy of the entire simulation cell, while *E*_polymer_ and *E*_CNT_ are potential energies of the isolated PEEK and CNT, respectively. The interaction energy between PEEK chains and CNT was calculated to be 7468 kcal/mol. The interfacial strength between the CNT and PEEK can be evaluated by their interfacial interaction. A strong interaction is desired for efficient load transfer between CNT and PEEK and the enhanced mechanical properties in the macro-scale. In addition, as no covalent chemical bonding is present in the system, the interfacial bond strength mainly comes from the van der Waals and electrostatic forces. The evolution of non-bonding interaction energy between PEEK and CNT is shown in Figure 12a. After the initial densification and equilibration of the system, a gradual decrease in the non-bonding potential energy is observed in the first 30 ns of the simulation, long after the neat PEEK reaches a steady-state response. The gradual decrease in the non-bonding energy indicates re-arrangement of the chains from an amorphous state to a partially ordered state that pulls down the energy level over time. Similar observations were made in the previous studies that perform simulations on polyethylene and alkane.^47,48^ The change in the orientational characteristics during this period is also verified in Figure 12c, where the mean value of the angle between PEEK chains’ backbone directions with respect to CNT is presented (Figure 12d). It is noted that all the simulations are performed close to the crystallization temperature which comes with advantages and disadvantages in terms of overcoming energy barriers to get different conformations. However, this effect is not expected to have a strong influence in PEEK, as there are no chiral centers and a tendency for cis and trans conformations.^49^Figure 12b shows the non-bonding contributions in the interactions between PEEK chains themselves with and without CNT. It is shown that the presence of CNT increases the interaction level between PEEK chains over time, which in part comes from the induced alignment and order in the system. This indicates that due to the strong van der Waals forces and ordered structure of PEEK chains accompanied by CNT, the PEEK chains around CNT also show a higher level of integrity.

**Figure 13:**
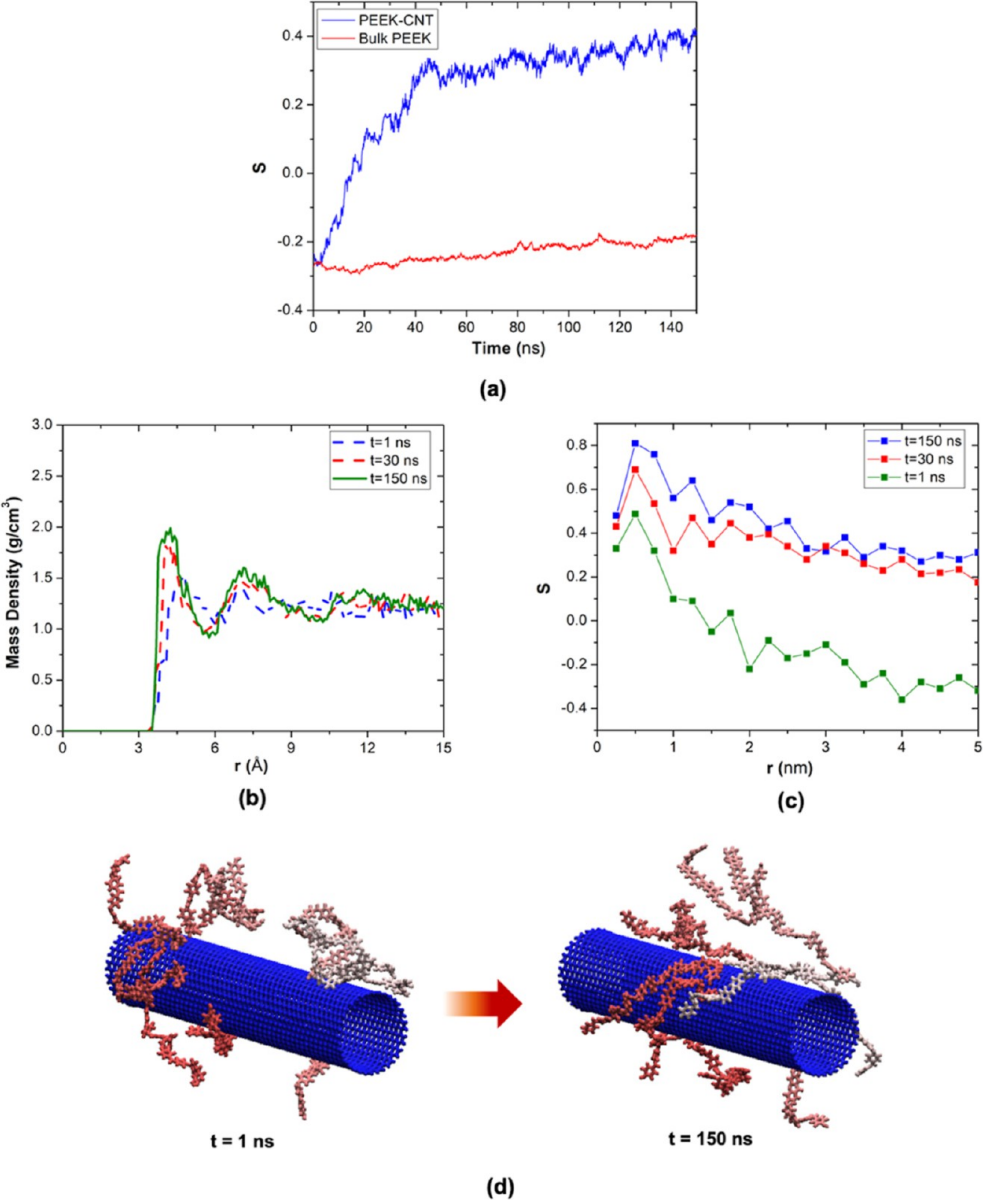
(a) Time evolution of the defined order parameter, *S*, for bulk PEEK and PEEK–CNT systems. (b) Mass density distribution of PEEK chains in the vicinity of CNT at three different times. (c) Radial distribution of the order parameter, *S*, for PEEK chains in snapshots taken after 1, 30, and 150 ns after simulation start. (d) Snapshots showing unfolding and elongation of 10 PEEK chains around CNT during simulations.

In order to understand the mechanism of change in the conformation of PEEK chains around CNT, the Herman’s orientation parameter, S, of the system is calculated as
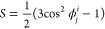
3where ϕ is the acute angle between the CNT axis and the fragment vector. It is noted that Herman’s parameter is also utilized in the above-mentioned azimuthal X-ray scans. As shown in Figure 12d, the fragment vector is generated by splitting the backbone of the PEEK chain into 13 fragments based on atom IDs. Obviously, valence angle fluctuations will affect the obtained angle distribution but not the system response form. The calculated order parameter *S* can have values in the interval [−0.5, 1]. *S* values close to 1 and −0.5 indicate chains perfectly parallel and perpendicular to the CNT axis, respectively. An *S* value close to zero indicates a random distribution in chain orientations. Figure 13a shows the time evolution of the order parameter, *S*, for PEEK chains over 150 nanoseconds, which is calculated by averaging the order parameters of all fragments, whose centers of mass are located within a 10 nm radial distance from the CNT. It is shown that the order in the PEEK–CNT system increases from random regimes into positive values that represent ordered chain conformation. It is evident that the major ordering appears in the initial 40 ns of the simulation; however, the uptrend keeps going by the end of the simulation, reaching *S* = 0.4. This is expected, as achieving a fully ordered molecular structure through all-atomistic simulations is not feasible. The PEEK system, on the other hand, experiences no considerable changes of conformation over 150 ns, keeping the random, amorphous characteristics. Figure 13d illustrates changes in the conformation of 10 randomly selected PEEK chains when they interact with the CNT. Chains tend to unfold and elongate along the CNT over time, which leads to an increase in the stiffness and strength of the nanocomposite in the CNT direction. The restricted movement of PEEK chains around CNT results in decreased chain entanglement. When the chains are less entangled with each other, they can establish a better-organized structure, promoting crystallinity.

Results show dynamics of structural formation of PEEK chains around CNTs from the amorphous phase toward the ordered structure, which demonstrates nucleation and growth of crystals induced by one-dimensional CNTs. This effect is responsible for maintaining the orientational features of the CNT-PEEK nanocomposite after rapid cooling, while the orientation in the bulk PEEK is lost after deposition, as demonstrated in SAXS and WAXS results.

To confirm the mechanism of change in the conformational order of polymer chains in the presence of CNT, the orientational characteristics of PEEK chains were analyzed with respect to the distance from the CNT surface in the radial direction. This was qualitatively characterized by calculating the order parameter S* for all the fragments of PEEK chains, whose centers of mass are located inside the parallel cylindrical mesh with an inner radius of *r* and outer radius of *r* + d*r*. The order parameter S* for each mesh is defined as
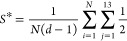
4where *N* and *d* are the number of PEEK chains in the mesh and the number of fragment vectors per chain, respectively. ϕ is the acute angle between the CNT axis and the fragment vector. Figure 13c shows the propagation of organized chains in the vicinity and farther away from the CNT over time. It is evident that CNT induces a considerable alignment in the neighboring chains even in the initial stages of the simulation at *t* = 1 ns; however, the chains farther away are in the random, amorphous regime. The responses at *t* = 30 ns and *t* = 150 ns indicate that the order propagates to the outer chains over time. It is noted that the Herman’s parameter obtained from SAXS and WAXS experiments is relatively lower, around 0.25, which is due to the difference in the scale of the regions under investigation. In the current simulation, the conformation of chains in the close vicinity of the CNT is investigated, which corresponds to the induced transcrystalline layers of PEEK, and is manifested by higher *S* values. The PEEK monomer has three aromatic rings and a carboxyl group that results in a stiff backbone structure; however, the absence of bulky pendant groups and dominance of sp^2^ bonds facilitate getting a planar conformation over time. Furthermore, it has been shown that polymers with a stiffer backbone show a more notable “memory effect”, which is demonstrated in the SAXS and WAXS data of the filaments and the printed roads.^50^

A similar meshing strategy was employed to calculate local atomic density around CNTs by assigning the atoms into shells according to their distances to the CNT. The density of each shell can be calculated by dividing the total mass of atoms by the volume of the shell. The confinement and ordering effect of CNT is also seen in the radial mass density curve of the system at different times, Figure 13b. It is evident that, besides high interaction levels, the promoted order in the vicinity of the CNT contributes toward forming denser packs of chains. In other words, as the chains unfold and elongate, they form a denser and more organized molecular arrangement. It is noted that the simulated bulk PEEK in this study has a density of 1.22 g/cm^3^, which differs about 5% from the corresponding experimental value of 1.27 g/cm^3^ for amorphous PEEK with no crystallinity.^51^

## 4 Conclusions

The use of nanocomposites in AM represents a vast opportunity space. The nanocomposite materials and AM process, in which they are used, need to complement each other to create higher quality parts for service. PEEK–CNT nanocomposite crystallinity was investigated in depth using WAXS, SAXS, and DSC techniques as well as all-atom MD simulations. Results suggest that crystallization is induced in the presence of CNTs during printing onto low-temperature build platforms (105 °C), while it is suppressed in the neat system. It has been shown that CNTs act as sites for crystallite nucleation and growth while simultaneously restricting polymer chain mobility and therefore % crystallinity in the material. Furthermore, the addition of the CNTs proved to be critical in the crystallization of FFF-printed roads, where the neat PEEK would be amorphous otherwise. The increased crystallinity in nanocomposite samples provided improved tensile strength and elastic modulus over the neat material. These observations were also verified through MD simulations, where the dynamics of nucleation is demonstrated, and it is shown that the presence of CNTs promotes the order in the PEEK chains and enhances the intermolecular interaction between the PEEK chains. It was concluded that the printed PEEK–CNT roads experience several phenomena that create these characteristics: (1) crystallite nucleation in the presence of CNTs, and (2) polymer chain confinement, resulting in the suppression of crystallization. The finding of crystallization in a rapid cooling environment through the addition of CNTs can be used to tailor the crystallinity of semicrystalline polymers and, consequently, optimize their mechanical properties. Further improvements to the use of PEEK–CNT in an FFF setting resulted in the minimization of the thermal gradient incurred during printing, which would improve the interlayer strength in the nanocomposites with higher CNT concentrations. Additionally, there is an opportunity to explore the cold crystallization phenomenon that occurs in the neat PEEK samples, which fosters the potential for shape memory or actuation of printed parts.

## Supporting Information Available

TGA and derivative curves, scanning electron microscopy imaging of nanocomposite filaments, and energy-dispersive spectroscopy images (PDF)
