# Supplementary material for: Fundamentals of Crystalline Evolution and Properties of Carbon Nanotube-Reinforced Polyether Ether Ketone Nanocomposites in Fused Filament Fabrication
Source: ACS Appl Mater Interfaces. 2023 Apr 26;15(18):22506–23. doi: 10.1021/acsami.3c01307 (PMC10853907; doi:10.1021/acsami.3c01307)
Supplement: Supplementary file 1 — am3c01307_si_001.pdf [file am3c01307_si_001.pdf]

Supporting Information

Fundamentals of Crystalline Evolution and Properties of Carbon Nanotube Reinforced Polyether Ether Ketone (PEEK) Nanocomposites in Fused Filament Fabrication (FFF)

*Mia Carrola[1§], Hamed Fallahi[2§], Hilmar Koerner[3], Lisa M. Pérez[4], Amir Asadi[1,2,5]**

1. Department of Materials Science & Engineering, Texas A&M University, College Station, Texas 77843, USA
2. Department of Mechanical Engineering, Texas A&M University, College Station, Texas 77843, USA
3. Materials & Manufacturing Directorate, Air Force Research Laboratory, WPAFB, Ohio 45430, USA
4. High Performance Research Computing, Texas A&M University, MS 3361, College Station, Texas 77843-3361, United States
5. Department of Engineering Technology & Industrial Distribution, Texas A&M University, College Station, Texas 77843

*Corresponding author: amir.asadi@tamu.edu

§ These authors contributed equally to this work

S1. Thermogravimetric Analysis

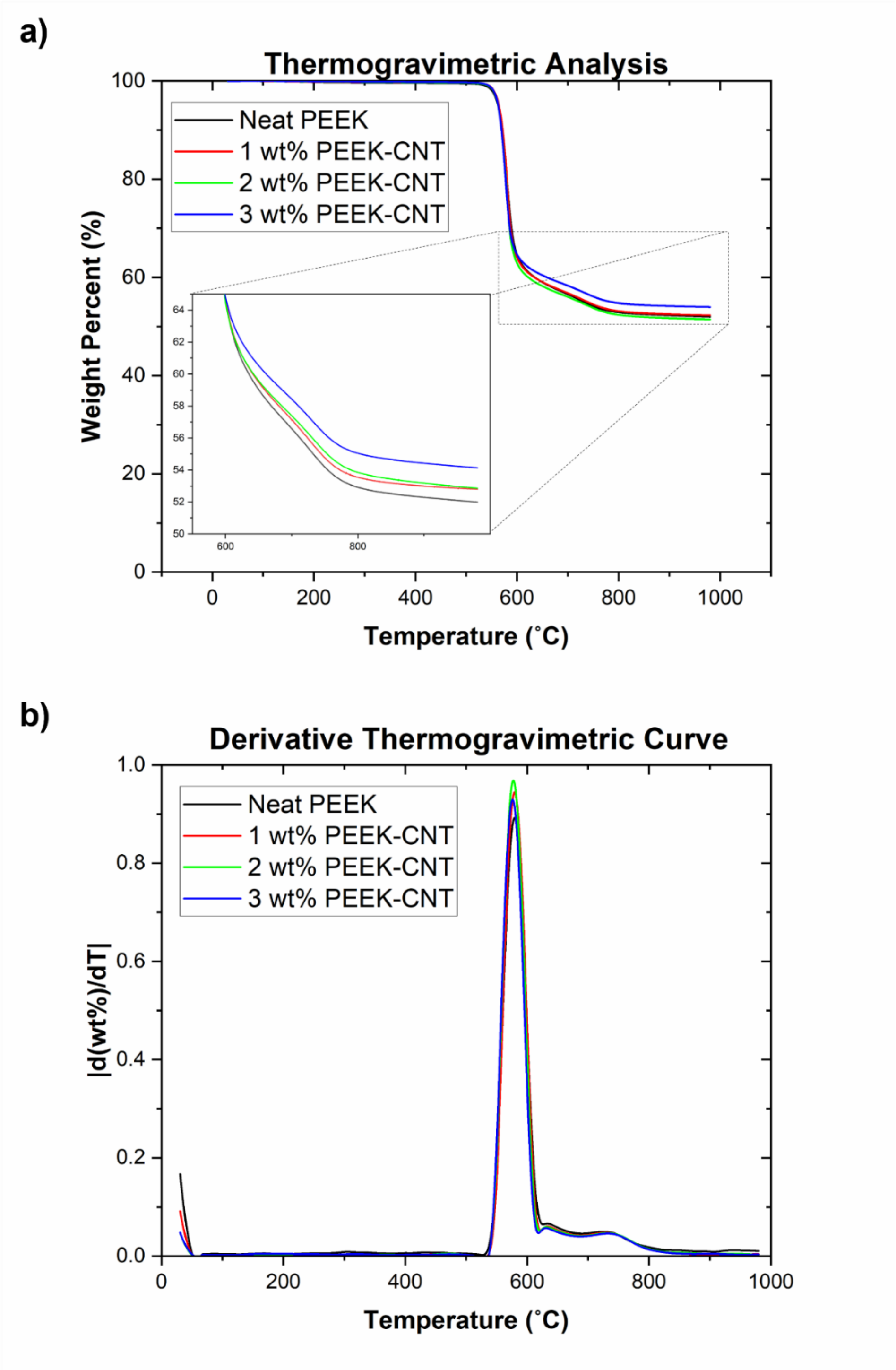

Figure S1. a) TGA and b) DTG curves that display the thermal stability of the nanocomposite materials compared to neat PEEK.

S2. SEM Images of Nanocomposite Filaments

SEM imaging was used in this study to evaluate both filaments and mechanical testing samples for voids, defects, fracture surfaces, and crack propagation behavior. The extruded PEEK-CNT filaments were imaged to examine if there were any air pockets or voids present within the feedstock, which could negatively affect the properties of printed parts. The four concentrations of filaments can be seen in Figure S2. There are no visible voids within any of the filament concentrations, which implies that any defects or voids that may be present in printed parts are resultant of the FFF process and not the initial filament extrusion. This eliminates the possibility of an influence stemming from the feedstock itself to the resultant printed parts, which aids in evaluating the effect that the AM method solely has on the material.

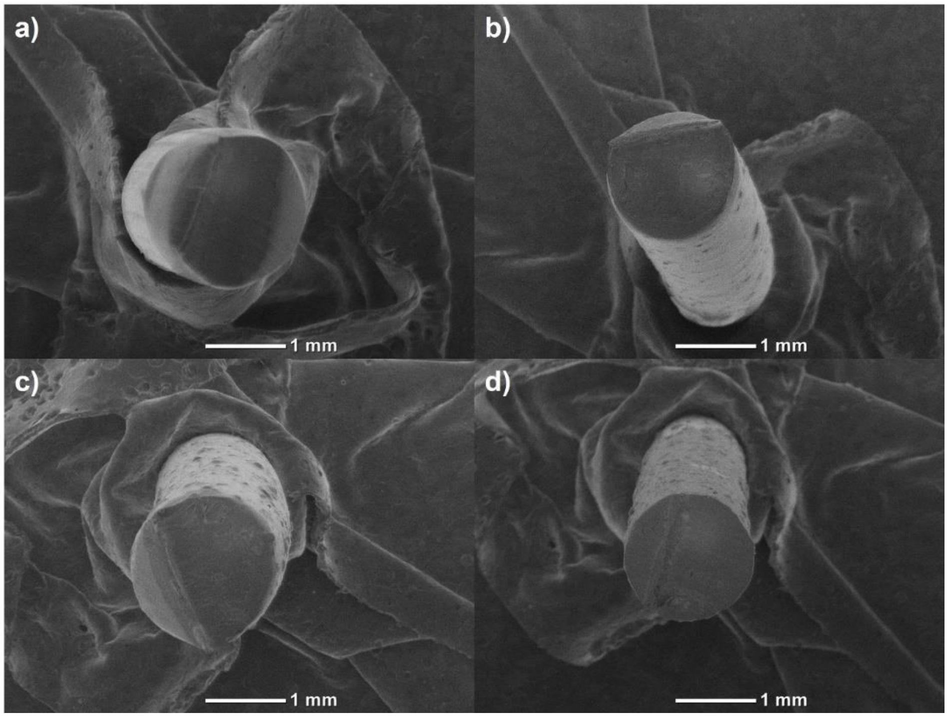

Figure S2. SEM images of the extruded nanocomposite filaments: a) neat PEEK, b) 1 wt% PEEK-CNT, c) 2 wt% PEEK-CNT, and d) 3 wt% PEEK-CNT. The images show no evidence of voids

within the feedstocks, implying that the imperfections seen in printed parts are from the FFF process itself.

S3. Energy Dispersive Spectrometry (EDS)

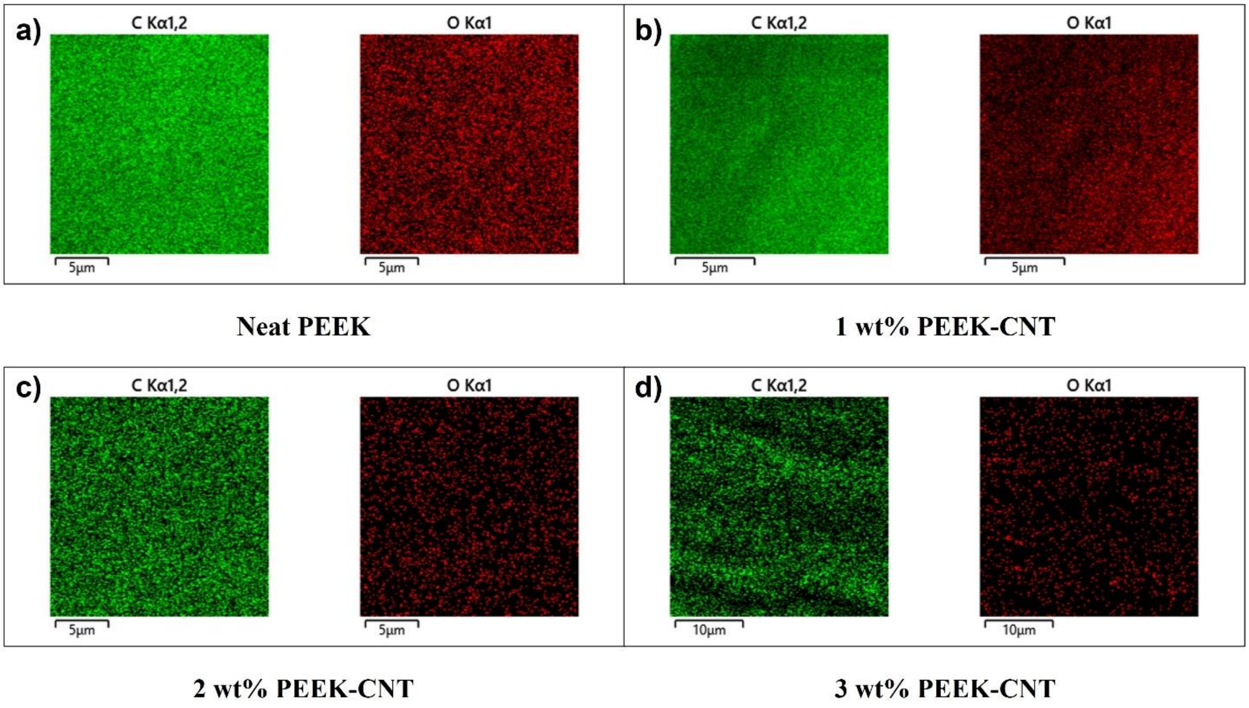

Figure S3: Energy dispersive spectrometry (EDS) mappings of a) neat PEEK, b) 1 wt% PEEK-CNT, c) 2 wt% PEEK-CNT, and d) 3 wt% PEEK-CNT showing carbon (green) and oxygen (red) atoms within printed samples. The oxygen atoms that are mapped can be interpreted as the PEEK itself, and the number of black empty areas (represented as additional black spots) begin to appear as representative of CNT additions.

Energy dispersive spectrometry (EDS) was utilized to evaluate the agglomeration and homogeneity within the printed material and can be seen in the figure above. Upon the addition of CNTs, the empty areas in the nanocomposite maps (represented as additional black spots) begin to appear and become more numerous as the concentration of CNTs increases. This is seen more

clearly in the mapping of the oxygen atoms, as the carbon atoms become more in quantity due to the fact that CNTs are composed of 95% carbon atoms according to the supplier and our previous XPS results[1]. Please note that we used pristine CNTs and not functionalized CNTs and therefore, there is no intentional oxygen containing groups grafted on CNTs. These observations suggest that the dispersion of the CNTs is relatively consistent throughout the printed sample. Though some agglomerations were present in printed samples shown in the SEM imaging, there is a homogenous dispersion of CNTs throughout the material according to the EDS mappings and TGA results.

References

1. Shariatnia, S., Kumar, A.V., Kaynan, O., and Asadi, A., Hybrid Cellulose Nanocrystal-Bonded Carbon Nanotubes/Carbon Fiber Polymer Composites for Structural Applications, *ACS Applied Nano Materials*, **3**(6):(2020) p. 5421-5436.
